# The mutational landscape of phosphorylation signaling in cancer

**DOI:** 10.1038/srep02651

**Published:** 2013-10-02

**Authors:** Jüri Reimand, Omar Wagih, Gary D. Bader

**Affiliations:** 1 The Donnelly Centre, University of Toronto, Canada

## Abstract

Somatic mutations in cancer genomes include drivers that provide selective advantages to tumor cells and passengers present due to genome instability. Discovery of pan-cancer drivers will help characterize biological systems important in multiple cancers and lead to development of better therapies. Driver genes are most often identified by their recurrent mutations across tumor samples. However, some mutations are more important for protein function than others. Thus considering the location of mutations with respect to functional protein sites can predict their mechanisms of action and improve the sensitivity of driver gene detection. Protein phosphorylation is a post-translational modification central to cancer biology and treatment, and frequently altered by driver mutations. Here we used our ActiveDriver method to analyze known phosphorylation sites mutated by single nucleotide variants (SNVs) in The Cancer Genome Atlas Research Network (TCGA) pan-cancer dataset of 3,185 genomes and 12 cancer types. Phosphorylation-related SNVs (pSNVs) occur in ~90% of tumors, show increased conservation and functional mutation impact compared to other protein-coding mutations, and are enriched in cancer genes and pathways. Gene-centric analysis found 150 known and candidate cancer genes with significant pSNV recurrence. Using a novel computational method, we predict that 29% of these mutations directly abolish phosphorylation or modify kinase target sites to rewire signaling pathways. This analysis shows that incorporation of information about protein signaling sites will improve computational pipelines for variant function prediction.

Cancer is a set of diseases characterized by somatically acquired cellular alterations that lead to selective advantages such as unrestricted growth, suppression of apoptosis and enhanced metabolism^1^. The complexity of cancer is observed at multiple levels of cellular organization, as somatic alterations in chromosomal copy numbers, epigenetic regulation and gene expression give rise to tumor types and subtypes with different biological and clinical properties^2^,^3^,^4^. In particular, high-throughput sequencing has revealed a complex landscape of somatic DNA mutations in cancer genomes^5^. Most cancer mutations are likely passengers that appear due to genetic, epigenetic and transcriptional instability, while few mutations, termed drivers, unlock oncogenic cell properties that lead to selective advantages and tumor development^1^,^6^. Cancer drivers are often discovered due to high mutation frequency across many tumors of a particular type, however combinations of rare mutations in related systems or pathways may be also responsible for tumorigenesis^7^,^60^. The accumulation of sequencing data from cancer genome projects^8^,^9^,^10^,^11^,^12^,^13^,^14^,^15^,^16^ now enables the discovery of driver mutations relevant across multiple tumor types. The characterization of these pan-cancer drivers is important for establishing efficient multi-cancer therapies such as the mutant BRAF inhibition strategy applicable in melanoma and leukemia^17^,^18^.

Cellular signaling networks are complex systems of interacting proteins that are ultimately encoded in the genome. Analysis of disease mutations using network context will thus lead to better understanding of their mechanisms of action^61^. Protein phosphorylation, a reversible post-translational modification (PTM) at serine (S), threonine (T) and tyrosine (Y) residues, involves a system of sequence-specific kinases (writers), phosphatases (erasers) and reader proteins. Phosphorylation signaling can modulate protein activity, alter protein folding, and help mediate or inhibit interactions with other proteins. Phosphorylation is important in cancer and is involved in the control of proliferation, oncogenic kinase signaling^19^, transcriptional regulation^20^, and TP53 activity^21^, among other processes. Phosphorylation is also a pharmacologically targetable mechanism with multiple approved therapies available for cancer treatment^17^,^18^. We recently proposed that cancer may be driven by statistically significant and spatially specific mutations in protein sites involved in cellular phosphorylation signaling, and developed the ActiveDriver method to detect such mutations comprehensively^7^. ActiveDriver is a gene-centric method that identifies signaling sites where the mutation rate is significantly higher than expected from the entire gene sequence, thus suggesting the site's importance in tumor biology.

The recently available pan-cancer dataset of 3,185 tumor genomes and 12 cancer types from The Cancer Genome Atlas (TCGA) comprises the largest collection of somatic cancer mutations to date^65^. It involves four times more samples and 24 times more SNVs than previous collections^7^, providing the opportunity to discover novel cancer driver genes across multiple cancer types. Here, we analyze the TCGA pan-cancer dataset of protein-coding missense single nucleotide variants (SNVs), as SNVs are easiest to interpret as specific alterations of signaling sites and are more reliably detected and abundant than other types of genetic mutations. We predict known and novel signaling-specific cancer driver genes, create a high-confidence collection of cancer mutations likely involved in altered cellular signaling, and propose numerous specific hypotheses to explain their functional effects.

## Results

### Signaling sites are important in mutation function prediction

**Figure 1:**
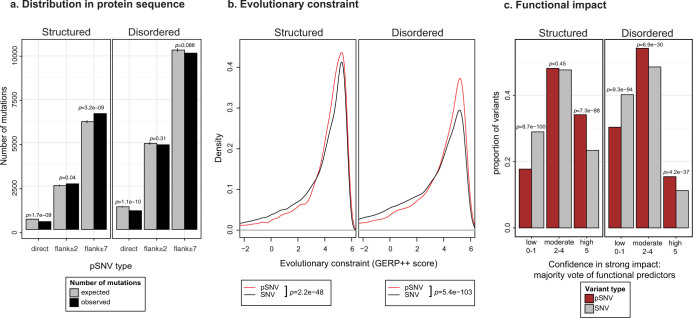
Properties of phosphorylation-related single nucleotide variants (pSNVs) in structured and disordered protein sequence. (a). Phosphosites in structured protein regions are significantly enriched in pSNVs (left panel), while disordered regions show no significant bias. Direct pSNVs altering phosphorylated residues are less common than expected. P-values are shown on top of bars (Poisson exact test). (b). Evolutionary constraint of pSNVs (red) is significantly greater than for other SNVs (grey) for structured as well as disordered protein sequence. Significance p-values are shown in the below plots (Wilcoxon test). (c). Prediction of variant functional impact with to five methods shows that pSNVs (red) are more frequently considered harmful by multiple methods. Predictor majority vote established confidence in functional impact, as low confidence (0–1 methods predict strong impact), moderate confidence (2–4 methods) and high confidence (5 methods). Bar height reflects proportion of variants. Significance p-values are shown above bars (binomial test).

To investigate cancer mutations in phosphorylation signaling, we collected 87,060 experimentally determined phosphosites in 10,185 human proteins and integrated these with 241,701 missense single nucleotide variants from the TCGA pan-cancer project (Supplementary Table 1). Including ±7 residues of phosphosite flanking regions and covering 7% of protein sequence, we found 16,840 phosphorylation-related SNVs (pSNVs) in 5,859 genes and 89% of all samples – over 17 times more pSNVs than previously discovered^7^ (Supplementary Tables 2–3). The observed large number of pSNVs helps us identify novel pan-cancer trends in phosphosite mutation distribution. We previously found that phosphosites are enriched in somatic mutations^7^. While this trend no longer holds with the larger pan-cancer dataset (Supplementary Fig. 1), we now identify a more specific signal – structured protein regions are enriched in pSNVs (*p* < 0.001, Poisson exact test), while disordered regions show no significant bias (Fig. 1a). pSNVs also show significantly stronger evolutionary constraint in 34 mammalian genomes^22^ compared to other SNVs (Fig. 1b), suggesting that these signaling sites undergo negative selection. Further, we compared the predicted functional impact of mutations in a majority vote^23^ of five state-of-the-art methods (SIFT^24^, PolyPhen2^25^, LRT^26^, PhyloP^27^, MutationTaster^28^) and found that pSNVs are more likely disruptive to protein function than other protein-coding mutations (Fig. 1c). According to another measure of pSNV importance, 1,427 direct pSNVs replace the central phosphorylated residue and thus disrupt phosphorylation; such mutations are under-represented on the whole, although frequently seen in known cancer genes such as *TP53* and *CTNNB1* (79 cancer genes, *p* = 4.2e − 18, Fisher's exact test). In total, we predict a specific signaling mechanism for 29% of pSNVs (4,800) through either direct pSNVs or kinase network rewiring (see below). Collectively, these global observations suggest that pSNVs represent a subset of protein-coding missense mutations with greater functional impact and relevance in cancer compared to other SNVs.

Interestingly, 40% of our mechanistic predictions (1,937 pSNVs) are considered neutral by most functional predictors, showing that incorporation of signaling information will greatly improve coverage of existing methods. We also observe that disordered regions comprising 37% of protein sequence carry the majority of phosphosites (71%) and thus represent an important but typically untreated factor in mutation analysis – one that ActiveDriver considers (Supplementary Fig. 2). While variants in disordered regions are generally less conserved than in structured regions, disordered pSNVs form a subset of higher evolutionary constraint and potential functional impact (Fig. 1b–c). In particular, 49% (977) of pSNVs in hallmark cancer genes occur in disordered regions. Thus, extending conservation-weighted functional mutation prediction methods to consider protein disorder will improve their coverage.

### ActiveDriver reveals pan-cancer driver genes with enriched pSNVs

**Figure 2:**
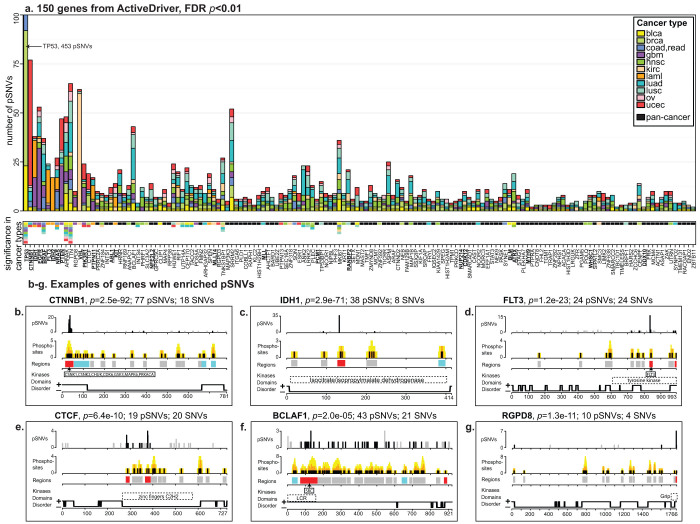
Genes with significant pSNVs in pan-cancer genomes. (a). ActiveDriver predictions of pan-cancer driver genes (*n* = 150, FDR *p* < 0.01). Top panel shows pSNV counts in pan-cancer genomes. Genes are ordered from left to right according to statistical significance, and colors indicate cancer types. Bottom panel shows summary of per-cancer pSNV analysis. Colored boxes show individual cancer types with pSNV enrichment, and black boxes represent genes found in pan-cancer data that were not significant in individual cancer types. Well-recognized cancer genes are in bold and underlined. (b–g). Examples of genes with enriched pSNVs. Six tracks of protein sequence information are shown, from top to bottom: (i) counts of pSNVs (black) and other SNVs (gray); (ii) location of phosphosites (black) and flanking regions (orange - within two amino acid positions, yellow - within 3–7 positions); (iii) ActiveDriver prediction of phosphosite regions as pSNV-enriched (red), pSNV-depleted (blue), and not significant (gray); (iv–v) kinases and protein domains associated to pSNV-enriched regions (LCR - low complexity region); (vi) regions corresponding to disordered (+) and ordered (−) protein sequence.

Next we used our ActiveDriver mutation significance model^7^ to predict a list of cancer driver genes specifically enriched in pSNVs. Pan-cancer analysis revealed 150 genes (FDR *p* < 0.01; Fig. 2a**, **Supplementary Table 4), many of which are known cancer genes (*n* = 26, *p* = 6.1e − 15, Fisher's exact test). Most genes (86), including seven known cancer genes, are only identified in the combined dataset but not in any individual cancer type, emphasizing the utility of integrated mutation analysis. Analysis of pSNVs recovers established driver mutations, validating our results, and provides novel signaling-related insight into functional consequence of many SNVs.

ActiveDriver reveals many known and novel cancer genes with specific pSNVs. For instance, beta-catenin (*CTNNB1*) is a Wnt-activated oncogene in lung and liver cancer that is degraded in non-tumor cells via phosphorylation of its N-terminus^20^. ActiveDriver identifies N-terminal mutations in five cancer types as highly significant (*n* = 73, FDR *p* = 2.5e − 92 from ActiveDriver; Fig. 2b), confirming constitutive activation of CTNNB1 via disrupted phosphorylation as a prevalent driving mechanism of cancer. As another example, isocitrate dehydrogenase 1 (IDH1) is a metabolic enzyme with frequent R132H mutations in leukemia and glioblastoma that associate to altered DNA methylation^29^. ActiveDriver suggests a novel hypothesis that 36 pSNVs modify the phosphosite Y135 recently observed in multiple proteomics datasets (Ref. 30, unpublished data at www.phosphosite.org; FDR *p* = 2.9e − 71; Fig. 2c). Kinase proteins are enriched in the predicted list of cancer drivers (*n* = 14, *p* = 1.0e − 07, Fisher's exact test), indicating that pSNVs also modify regulators in signaling networks. The tyrosine kinases FLT3^19^ (Fig. 2d) and KIT^31^ are the most prominent examples of significant activation loop pSNVs in leukemia that lead to increased kinase activity and acquired drug resistance, while we also observe similar mutations in other kinases, such as HCK and CHEK2. In addition to oncogenic kinases, pSNVs also modify transcription factors (TFs). For instance, the multifunctional TF and candidate tumor suppressor CTCF is differentially phosphorylated in its DNA-binding domains during cell cycle^32^,^33^. ActiveDriver highlights 19 pSNVs at these sites in six cancer types (FDR *p* = 6.4e − 10; Fig. 2e), providing phospho-mechanistic insight into the earlier observation that cancer mutations in CTCF DNA-binding domains alter transcription of proliferative genes^34^. The candidate cancer gene BCLAF1 (Bcl-2-associated transcription factor 1) is another phosphorylated TF with frequent pSNVs in sites of SRC onco-kinase (Fig. 2f). Finally, *RGPD8* is an example of a poorly characterized gene with recurrent and specific pSNVs of four cancer types in the Grip domain related to Golgi signaling and protein secretion (Fig. 2g). In summary, analysis of phospho-mutations in cancer helps predict novel cancer driver genes and signaling-related mechanisms for known cancer genes.

### Gain-of-signaling and loss-of-signaling pSNVs cause oncogenic rewiring of the kinase network

**Figure 3:**
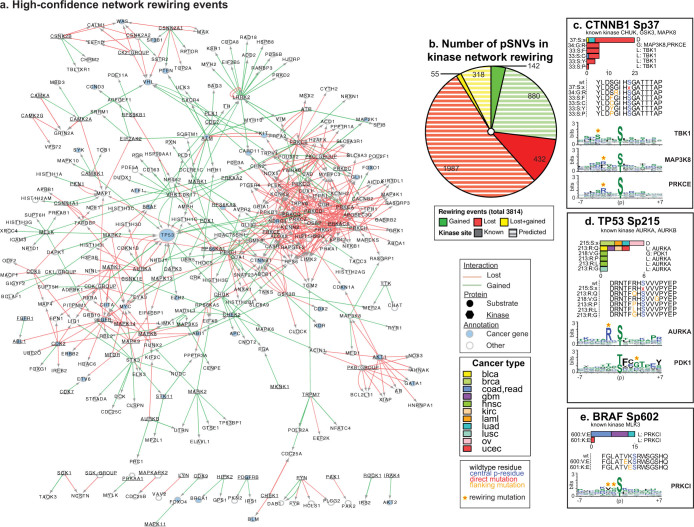
pSNVs in kinase network rewiring. (a). The high-confidence network of gain-of-signalling (green edges) and loss-of-signaling events (red edges) induced by pSNVs was predicted for 96 kinase binding models (*p* < 0.05). Node size corresponds to number of network-rewiring pSNVs, and edge weight shows predicted impact of pSNV to kinase binding. Known cancer genes are shown as blue nodes and kinases are underlined. (b). Total number of pSNVs predicted to induce network rewiring (red – loss-of-signaling; green – gain-of-signaling; yellow – switch-of-signaling). pSNVs affecting known kinases (solid colors) and predicted kinases (striped colors) are shown separately. (c–e). Examples of phosphosites with network-rewiring pSNVs. Each panel contains three plots from top to bottom. (i) Bar chart shows pSNV counts in different cancer types and rewiring events for kinases (L – loss; G – gain; D – direct pSNV). Phosphosite and its experimentally determined kinases are shown on top. (ii) Block of 15-mer peptides shows pSNVs in phosphosite sequences (wildtype (wt) amino acid – black; central residue – blue; flanking pSNV – orange; direct pSNV – red). (iii) Predicted kinase binding models as position weight matrices (PWMs), with phosphosite sequence on X-axis (±7 residues, central residue indicated with ‘p’), and information content on Y-axis. Letter height corresponds to prevalence of amino acid at given position. Red asterisks in kinase binding models denote residues affected by network-rewiring pSNVs.

As most pSNVs occur in phosphosite flanking sequence, we sought to characterize their impact on kinase binding specificity, which may affect site phosphorylation. We first developed a high-confidence set of sequence patterns recognized by 96 kinases modeled as position weight matrices (PWMs), based on known kinase binding sites (Supplementary Fig. 3). We next predicted which kinases recognize known phosphosites and how this changes after pSNV mutation. We identified 3,814 significant network-rewiring mutations – losses or gains in kinase-substrate signaling – that alter 11,802 potential kinase-substrate interactions and cover 23% of all pSNVs (*p* < 0.05; Supplementary Table 5). A high-confidence network includes 392 pSNVs in 534 interactions, and comprises only top-scoring kinase binding sites for signaling gain and experimentally determined kinase-substrate signaling loss (Fig. 3a). Most inferred network-rewiring pSNVs confer loss-of-signaling, but many added interactions and change-of-signaling events are also predicted (Fig. 3b). Several examples of well-studied mutations are apparent in the rewiring analysis, which help validate our results. N-terminal phosphorylation of CTNNB1 at S37 involves the most frequent rewiring with 23 direct pSNVs and 23 flanking pSNVs (Fig. 3c) leading to predictions of gained signaling (with MAP3K8, PRKCE kinases) and lost signaling (TBK1). Notably, the gain-of-signaling mutation G34R may involve Wnt-MAPK signaling of CTNNB1 activation^35^, or the protein kinase C pathway that degrades CTNNB1 independently of Wnt^36^. As another example, phosphorylation of TP53 by AURKA kinase at S215 is known to inhibit TP53 tumor suppression function^21^. We predict that AURKA-TP53 signaling at that site is disrupted by six direct and seven flanking pSNVs (Fig. 3d), leading to increased activity of TP53. This potentially explains our earlier observation of improved survival of ovarian cancer patients with TP53 pSNVs^7^ and encourages further investigation of the site as a prognostic biomarker. Next, our analysis predicts that the BRAF V600E kinase activation mutation^37^ implicated in therapy of melanoma^17^ and leukemia^18^ interferes with phosphorylation by PRKCI at S602 (Fig. 3e). Phosphorylation of wildtype BRAF at S602 by MLK3 is known to activate cell proliferation pathways, however BRAF V600E tumor cells proliferate independently of MLK3^38^. We therefore speculate that BRAF signaling may involve PRKCI or another unrecognized kinase in a combinatorial or competitive mechanism with MLK3. Such kinase binding site analysis shows that flanking pSNVs may frequently lead to oncogenic rewiring of signaling networks, thus identifying specific mechanistic hypotheses about cancer-driving mutations.

### Mutated signaling is central to hallmark cancer pathways and transcription regulatory networks

**Figure 4:**
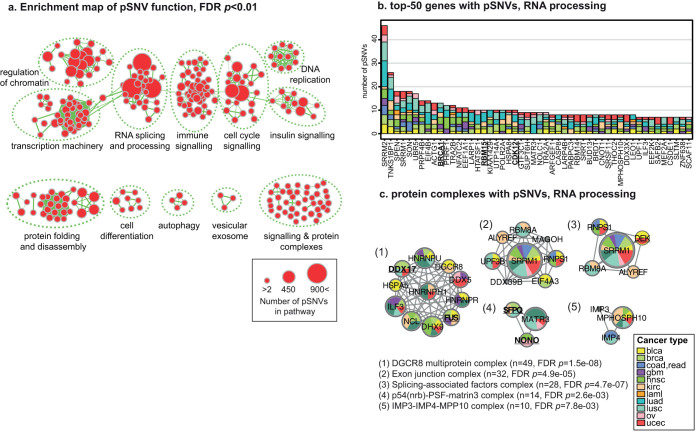
Pathways and processes with enriched pSNVs. (a). Enrichment map of over-represented functional themes for pan-cancer pSNVs (FDR *p* < 0.01). Functional enrichments also apparent in global SNV analysis have been filtered. Nodes represent pathways and processes, and edges show overlap of corresponding gene sets. Major functional themes are circled and annotated. (b). Top-50 RNA splicing and processing genes with frequent pSNVs. Colors denote cancer types. (c). Protein complexes with enriched pSNVs from the theme of RNA splicing and processing. Nodes correspond to complex subunits, and node size corresponds to pSNV count. Known cancer genes are in bold and underlined.

Cancer is a disease of pathways driven by systematic alterations in hallmark processes^1^. Thus many infrequent but specific pSNVs may affect different components of underlying systems and lead to tumorigenic cell properties. To establish the significance of altered signaling at the pathway level, we conducted a functional enrichment analysis of somatic mutations. We focused on a stringently filtered subset of pathways specifically enriched in pSNVs and not in other SNVs, and revealed multiple phosphorylation-related functional themes with pan-cancer significance (FDR *p* < 0.01, Poisson exact test; Fig. 4a, Supplementary Fig. 4**, **Supplementary Table 6). The major functional themes are related to regulation of gene expression on epigenetic, transcriptional and post-transcriptional levels, cell cycle and differentiation, and immune and insulin signaling. For example, the RNA splicing and processing theme involves 2,009 pSNVs in 652 genes (Fig. 4b), including 38 known cancer genes such as *BRCA1*, *RBM15*, *CDK12*, *CDC73* and *TOP1* (FDR *p* = 1.3e − 06, Fisher's exact test), as well as candidate cancer genes. For instance, the transcription factor SPEN, a negative regulator of Notch proliferative signaling pathway^39^, has 19 pSNVs in ten cancer types (FDR *p* = 2.2e − 03 from ActiveDriver). Enrichment analysis also highlights pSNVs in protein complexes of RNA regulation (Fig. 4c), such as the DGCR8 multiprotein complex^40^ of microRNA processing (49 pSNVs) and the exon junction complex^41^ of mRNA splicing and post-transcriptional regulation (32 pSNVs).

**Figure 5:**
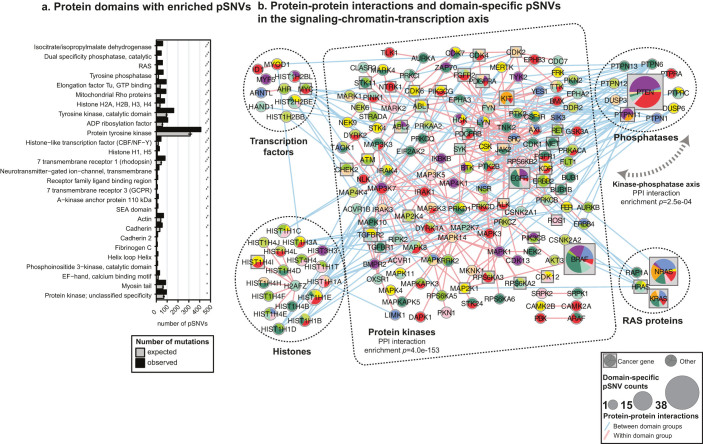
pSNVs in structural protein domains. (a). SMART and Pfam protein domains with significant enrichment of phosphosite mutations (FDR *p* < 0.05; domains with ≥25 pSNVs shown). Asterisks indicate level of statistical significance. (b). Protein-protein interaction (PPI) network of proteins with significant enrichment of domain-specific pSNVs. Nodes are grouped by domain type, as kinases (phospho-writers, center), phosphatases (phospho-erasers; top right), histones (chromatin regulation; bottom left), transcription factors (TFs, regulation of gene expression; top left), and RAS proteins (signaling, bottom right). Edge color denotes type of interaction (red – interaction within domain-specific group; blue – interaction between groups). Node size corresponds to number of domain-specific pSNVs, and node coloring reflects cancer types. Significant enrichment of PPIs is observed within the kinase group and in the kinase-phosphatase axis (p-values, Poisson exact test).

As oncogenic signaling mutations appear in central gene regulatory processes, we hypothesized that pSNVs alter interactions and activity of regulatory protein domains. To investigate this in detail, we studied 5,662 protein domains for specific pSNV enrichment, and found 27 unique domains with frequent signaling mutations (≥25 pSNVs, FDR *p* < 0.05, Poisson exact test, Fig. 5a, Supplementary Table 7). pSNVs are most abundant in kinase domains (446 pSNVs in 197 kinases), as kinases are regulated by phosphorylation in complex hierarchical networks^7^. In addition, pSNV enrichment is apparent in RAS, phosphatase, histone, and transcription regulatory domains. Interestingly, neurotransmitter genes such as GABA receptors were enriched in pSNVs in ligand-binding and transmembrane domains. Protein-protein interaction (PPI) network analysis of domain-specific pSNVs emphasizes the centrality of altered kinase signaling (Fig. 5b, Supplementary Table 8). In particular, the group of kinase proteins involves more within-group PPIs than expected (*n* = 253, *p* = 4.0e − 153, Poisson exact test), and the phosphorylation reader-writer system of kinases and phosphatases is also highly interacting (*n* = 52, *p* = 2.5e − 04). The domain-centric network includes 499 specific pSNVs in 183 proteins, including 42 known cancer genes (*p* = 1.5e − 28, Fisher's exact test). In summary, pathway and domain enrichment analysis shows that multiple regulatory systems and novel cancer hallmark processes are extensively altered by specific, potentially targetable mutations in phosphorylation signaling.

## Discussion

Our analysis demonstrates the extent of signaling-related mutations in a dozen important cancer types. A quarter of pSNVs are predicted to directly disrupt phosphorylation or involve kinase rewiring at the sequence level.

Our developed mechanistic hypotheses potentially depend on many factors of the cellular context. For instance, when observing a mutated signaling site, we assume that the site is actually phosphorylated in the tumor of study. While this information cannot be confirmed in general, we rely on the principle of evolutionary selection in cancer to gain confidence in our predictions. A major indicator of cancer driver mutations is their frequent, statistically significant recurrence in multiple tumor samples. The observation of repeated random events in precisely the same genomic locus is statistically so unlikely that we instead suggest that the site is under positive selection due to its role in tumor growth. The stronger the observed selection signal, the higher our confidence in calling the gene a cancer driver, regardless of our lack of knowledge about the context of the expressed protein in tumor cells. This is a standard and powerful approach in the cancer genomics field.

One limitation of the current analysis is that we restrict observed signaling alterations to protein-coding SNVs that comprise a minority of all cancer mutations known to affect protein function. We are thus underestimating the extent of mutated signaling in tumor cells caused by other mechanisms, for example genomic deletions such as the EGFRvIII isoform in glioblastoma, or translocations such as BCR-ABL in leukemia. However, the specific focus on protein-coding variants provides us with a high-confidence set of mutations that are simple to interpret in the context of cell signaling and lead to novel hypotheses that can be experimentally tested. In particular, tumor cell lines can be infected with mutated proteins using lentiviral technology, and phosphorylation-specific antibodies and mass spectrometry can be used to compare site-specific phosphorylation in mutated cell lines and controls. The functional consequence of such mutations can be characterised in phenotypic readouts such as growth assays or drug response screens. The impact of mutations on kinase binding specificity can be explored in in vitro protein-binding microarrays, or in vivo, for example, by measuring kinase target phosphorylation after kinase mutation.

We previously performed ActiveDriver analysis on 800 cancer samples of eight cancer types^7^. Here we extend our analysis to 3,185 tumor genomes and 12 cancer types. As a result we predict 54 additional cancer-specific drivers and 82 genes only seen in pan-cancer analysis. In a related analysis, we combined these ActiveDriver results with predictions from MuSiC^62^, OncodriveFM^63^, and OncoDriveClust^64^ to establish a high-confidence set of pan-cancer driver genes^66^.

The major goal of our analysis is to mechanistically explain the impact of phospho-mutations. Previously we could only specify that mutation of the central S, T, or Y amino acid to a non-STY residue would cause a loss of signaling. Here, we introduce a novel method to predict cancer-specific kinase rewiring events that considers how mutations in the phosphosite flanking region affect protein function. This approach increases the coverage of potential mechanistic explanations from 8% to 29% of all pSNVs. For our kinase rewiring analysis, we only cover 20% of human kinases and do not include information about kinase expression in a given tumor sample. Additional kinase specificity and tumor-specific protein expression data will improve the coverage and accuracy of these predictions.

In summary, this analysis leads to three major conclusions. First, incorporation of signaling information improves the sensitivity and coverage of variant function prediction and helps discovery of cancer driver mutations. Second, protein disorder is an important factor that functional mutation prediction methods should account for. Third, somatic alteration in phosphorylation signaling is a pan-cancer phenomenon, at least in the 12 cancer types studied here. As phosphorylation is a targetable mechanism with proven rational agents such as kinase inhibitors, consideration of pan-cancer pSNVs in therapy development may help find additional treatment strategies applicable to multiple cancer types.

## Methods

### Protein data

Sequences for completed human refGene genes (hg19) were translated to proteins with the Annovar toolset for functional annotation of genetic variants^42^ and filtered to retain the longest isoform for every gene. Pseudo-autosomal genes and genes with non-standard chromosomal annotations were discarded. Protein disorder was predicted with the DISOPRED2 toolset^43^ using default parameters. Experimentally validated and published phosphorylation sites in human proteins were retrieved from three databases (HPRD^44^, PhosphositePlus^45^, Phospho.ELM^46^). Phosphorylated sites with ±7 residues were matched exactly to the longest isoforms of annotated proteins, allowing multiple matches per sequence. Sites with overlapping flanking sequences were merged into continuous regions. SMART^46^ and Pfam^47^ protein domains were predicted with the SMART webservice. Physical human protein-protein interactions (PPI) were retrieved from the BioGRID database^48^ and auto-interactions were discarded. The list of 555 canonical cancer genes was compiled from earlier review papers^6^,^49^,^50^,^51^ using the CancerGenes^52^ and Cancer Gene Census^6^ databases.

### Mutation data

The cleaned and filtered pan-cancer mutation annotation file for 12 cancer types was retrieved from The Synapse database (www.synapse.org; ID syn1729383). Hypermutated samples (71) were discarded. DNA mutations were translated to protein sequence with Annovar^42^. Only missense single nucleotide variants (SNVs) were retained and other mutation types including stop codon mutations were discarded. Mutations were mapped to phosphosites with ActiveDriver^7^. pSNVs were classified as direct (mutation at central phosphorylated residue, S/T/Y), proximal flanking (mutation within 2 residues of the central residue), and distal flanking (mutation within 7 residues). The set of direct mutations was further filtered −105 serine-threonine mutations (S > T, T > S) were not considered direct, as these are known to be equivalent in terms of kinase specificify. Phosphosite mutations and other supplementary data accompanying our analysis are stored in the Synapse database (www.synapse.org, ID syn2237931).

### Statistical analysis of pSNVs

The distribution of SNVs with respect to phosphosites was assessed with the Poisson exact test by comparing the mutation frequencies (average number of SNVs per residue) in phosphosite-related and non-related sequence, using all proteins in the dataset. Robust expected range of pSNVs was sampled from the Poisson distribution (median ± median absolute deviation). Distribution of phosphosites in disordered and structured regions was assessed similarly with background mutation rates estimated from disordered and structured sequence, respectively. GERP++ scores for evolutionary constraint of mutations in human and 33 other mammalian species^22^, as well as functional predictions from five methods (SIFT^24^, PolyPhen2^25^, LRT^25^, PhyloP^27^, MutationTaster^28^) were retrieved from Annovar^42^. Evolutionary constraint of pSNVs and other SNVs was compared with the non-parametric Wilcoxon test as well as custom permutation tests (*p* < 1e − 6). Prediction of functional impact of mutations was carried out as a majority vote of the five methods, using the cutoff criteria as defined in the dbNSFP database of human non-synonymous SNPs^23^. Each mutation was scored by how many methods considered it harmful, and was binned as low (0–1), moderate (2–4) and high confidence (5), as previously proposed^23^. Statistical significance of pSNVs in bins was assessed with the binomial test using bin frequencies of other SNVs as background. Significantly phospho-mutated genes were computed with ActiveDriver^7^ (false discovery rate (FDR) *p* < 0.01). Two genes with significance only from depleted pSNVs were discarded (PIK3CA, KRAS).

### Kinase sequence binding models

Kinase amino acid binding specificities were modeled as position weight matrices (PWMs) with 15 positions. PWM columns represent sequence positions, rows represent amino acids, and values represent probabilities of amino acids being present in a particular position of a kinase binding site. Initial PWMs were constructed from 15,659 sequence-specific kinase binding site annotations collected from HPRD, PhosphositePlus and Phospho.ELM. Kinase models with less than 20 binding sequences were discarded. PWMs were then refined using a strategy that discarded outlier sequences (cutoff *p* = 0.2) from each kinase-specific sequence set and constructed the PWM iteratively until convergence (no further sequences below cutoff). PWMs were discarded if the refining procedure provided less than 20 sequences as result. Refined PWMs were assessed in tenfold cross-validation experiments in which 80% of known refined kinase binding sequences were used for PWM construction and the remaining 20% served as the positive test set. The negative test set for validation using target sequences from other kinases. Area Under Receiver Operator Curve (AUROC) statistics were used for PWM evaluation and low-quality PWMs were discarded (*AUROC* < 0.75). The remaining high-confidence 96 PWMs (Supplementary Fig. 3) corresponding to 7,606 sequences were used to evaluate pSNVs impact on kinase binding to phosphosites.

### Analysis of kinase network rewiring

To assess the similarity between a given sequence and a PWM, we adopted the Matrix Similarity Score (MSS) of the MATCH DNA binding model^53^ to amino acids. We excluded the central S/T/Y residue (column 8 in PWM) from scoring to focus on flanking sequences. To establish the statistical significance of MSS values for a particular PWM, we computed an empirical background distribution of MSS scores. The background comprised all phosphosites with known kinases that were not associated to the kinase of the particular PWM. The significance p-value of a given MSS score was determined as the fraction of background sequences with equal or greater MSS. Impact of mutations on kinase binding was assessed using empirical p-values. A pSNV was considered to lead to loss-of-signaling of a kinase if a given reference phosphosite had a significant p-value of MSS (*pRef* < 0.05) and the corresponding mutated phosphosite had a non-significant MSS p-value (*pMut* > 0.1) for the PWM of that kinase. Gain-of-signaling pSNVs were predicted similarly (*pRef* > 0.1 and *pMut* < 0.05 for a PWM). A pSNV was considered to rewire a binding site (lead to switch-of-signaling) if both gain and loss events with different PWMs were predicted for the same pSNV.

### Protein domain analysis

5,662 protein domains from SMART and Pfam databases were matched to human protein sequences. Infrequent domains (<3 proteins) and infrequently mutated domains (<3 proteins with domain-specific pSNVs) were filtered. The remaining 525 domains were tested for pSNV enrichment with one-tailed Poisson exact tests, by comparing domain-specific pSNV counts in domain-associated sequence with expected mutation rate of all phosphorylation-associated protein sequence. Results were filtered for significance (FDR *p* < 0.05) and further filtered manually for redundancy. The domain network comprises protein-protein interactions (PPI) between proteins with significantly phospho-mutated domains. Proteins were grouped by domain type (kinases - TyrKc, STYKc, PI3Kc, Pkinase_Tyr; phosphatases - Pfam:DSPc, Pfam:Y_phosphatase; histones - Pfam:Histone, Pfam:Linker_histone; transcription factors - Pfam:HLH, Pfam:CBFD_NFYB_HMF) and assessed for enrichment of PPI within groups and between groups using the one-tailed Poisson exact test. The test considered number of interactions for a list of proteins (e.g. interactions within a list of phospho-mutated proteins with kinase domains), given the average number of interactions for all proteins in our set.

### Pathway enrichment analysis

Functional gene lists representing pathways and processes were retrieved from the g:Profiler web server^54^ and filtered to exclude large lists (>1,000 genes) and small lists (<3 genes). Gene Ontology^55^ terms, Reactome^56^ pathways and protein complexes from the CORUM database^57^ were used for enrichment tests and other data sources were discarded. Gene lists with mutations in only single genes or samples were also discarded. Pathway enrichment analysis of mutations in 14,135 processes, complexes and pathways was carried out with one-tailed Poisson tests. Separate pathway analyses were carried out for SNVs and pSNVs, and both mutation groups were tested for cancer type-specific and pan-cancer pathway enrichments. Multiple testing corrections with FDR were applied separately for each data source (GO, Reactome, CORUM) mutation type (pSNV, SNV), and cancer type (12 cancer types plus pan-cancer dataset). The tests compared number of mutations within a functional gene list, given the average number of mutations across all genes (SNV analysis) or across genes with phosphosites (pSNV analysis). The final list of significantly mutated phospho-specific pan-cancer pathways included results from the pan-cancer analysis (FDR *p* < 0.01) that were not significant in the SNV analysis of individual cancer types (FDR *p* > 0.1) or the SNV analysis of the pan-cancer dataset (FDR *p* > 0.1). Processes and pathways were visualized as Enrichment Maps^58^ with the Cytoscape software^59^.

## Author Contributions

J.R. designed the study, performed data analysis and interpretation, prepared figures and wrote the manuscript. O.W. led analysis of kinase network rewiring, and designed and implemented the related method. G.D.B. supervised the study and wrote the manuscript. All authors helped finalize the manuscript and approved the final version.

## Supplementary Material

Supplementary Information Supplementary Note 1

Supplementary Information Supplementary Tables 1–8

Supplementary Information Supplementary Information
