# Supplementary material for: The mutational landscape of phosphorylation signaling in cancer
Source: Sci Rep. 2013 Oct 2;3:2651. doi: 10.1038/srep02651 (PMC3788619; doi:10.1038/srep02651)
Supplement: Supplementary Information — Supplementary Note 1 [file srep02651-s1.pdf]

Salutation	First Name	Last Name	TCGA Component (Institute, center type)	Email Address	Nature ID (optional)	Institution #1 (address, zip code)	Institution #2 (address, zipcode)	Institution #3 (address, zip code)
Mr.	Kyle	Chang	Baylor, GCC	kyleyk02@gmail.com		Human Genome Sequencing Center, Baylor College of Medicine, Houston, TX 77030		
Dr.	Chad J.	Creighton	Baylor, GCC	creighto@bcm.edu		Human Genome Sequencing Center, Baylor College of Medicine, Houston, TX 77030		
Dr.	Caleb	Davis	Baylor, GCC	cdavis@bcm.edu		Human Genome Sequencing Center, Baylor College of Medicine, Houston, TX 77030		
Dr.	Lawrence	Donehower	Baylor, GCC	larryd@bcm.edu		Human Genome Sequencing Center, Baylor College of Medicine, Houston, TX 77030		
Ms.	Jennifer	Drummond	Baylor, GCC	jenn@bcm.edu		Human Genome Sequencing Center, Baylor College of Medicine, Houston, TX 77030		
Dr.	David	Wheeler	Baylor, GCC	wheeler@bcm.edu		Human Genome Sequencing Center, Baylor College of Medicine, Houston, TX 77030		
Ms.	Huyen	Dinh	Baylor, GSC	hdinh@bcm.edu		Human Genome Sequencing Center, Baylor College of Medicine, Houston, TX 77030		
Dr.	HarshaVardhan	Doddapaneni	Baylor, GSC	doddapan@bcm.edu		Human Genome Sequencing Center, Baylor College of Medicine, Houston, TX 77030		
Dr.	Richard	Gibbs	Baylor, GSC	agibbs@bcm.edu		Human Genome Sequencing Center, Baylor College of Medicine, Houston, TX 77030		
Dr.	Preethi	Gunaratne	Baylor, GSC	preethig@bcm.edu		Human Genome Sequencing Center, Baylor College of Medicine, Houston, TX 77030	Department of Biology and Biochemistry, University of Houston, Houston TX 77204	
Dr.	Yi	Han	Baylor, GSC	yhan@bcm.edu		Human Genome Sequencing Center, Baylor College of Medicine, Houston, TX 77030		
Ms.	Divya	Kalra	Baylor, GSC	divyak@bcm.edu		Human Genome Sequencing Center, Baylor College of Medicine, Houston, TX 77030		
Ms.	Christie	Kovar	Baylor, GSC	ckovar@bcm.edu		Human Genome Sequencing Center, Baylor College of Medicine, Houston, TX 77030		
Ms.	Lora	Lewis	Baylor, GSC	lpampell@bcm.edu		Human Genome Sequencing Center, Baylor College of Medicine, Houston, TX 77030		
Ms.	Margaret	Morgan	Baylor, GSC	mmorgan@bcm.edu		Human Genome Sequencing Center, Baylor College of Medicine, Houston, TX 77030		
Ms.	Donna	Morton	Baylor, GSC	dvilla@bcm.edu		Human Genome Sequencing Center, Baylor College of Medicine, Houston, TX 77030		
Ms.	Donna	Muzny	Baylor, GSC	donnam@bcm.edu		Human Genome Sequencing Center, Baylor College of Medicine, Houston, TX 77030		
Dr.	Jeffrey	Reid	Baylor, GSC	jgreid@bcm.edu		Human Genome Sequencing Center, Baylor College of Medicine, Houston, TX 77030		
Dr.	Min	Wang	Baylor, GSC	mwang@bcm.edu		Human Genome Sequencing Center, Baylor College of Medicine, Houston, TX 77030		
Ms.	Liu	Xi	Baylor, GSC	lxi@bcm.edu		Human Genome Sequencing Center, Baylor College of Medicine, Houston, TX 77030		
Mr.	Adrian	Ally	BC Cancer Agency, GCC	aally@bcgsc.ca		Canada's Michael Smith Genome Sciences Centre, BC Cancer Agency, Vancouver, BC V5Z 4S6		
Ms.	Miruna	Balasundaram	BC Cancer Agency, GCC	mbala@bcgsc.ca		Canada's Michael Smith Genome Sciences Centre, BC Cancer Agency, Vancouver, BC V5Z 4S6		
Dr.	Inanc	Birol	BC Cancer Agency, GCC	ibirol@bcgsc.ca	ibirol	Canada's Michael Smith Genome Sciences Centre, BC Cancer Agency, Vancouver, BC V5Z 4S6	Department of Medical Genetics, University of British Columbia, Vancouver, BC V6H 3N1	School of Computing Science, Simon Fraser University, Burnaby, BC V5A 1S6
Mr.	Yaron S.N.	Butterfield	BC Cancer Agency, GCC	ybutterf@bcgsc.ca		Canada's Michael Smith Genome Sciences Centre, BC Cancer Agency, Vancouver, BC V5Z 4S6		
Mr.	Andy	Chu	BC Cancer Agency, GCC	achu@bcgsc.ca	achu	Canada's Michael Smith Genome Sciences Centre, BC Cancer Agency, Vancouver, BC V5Z 4S6		
Mr.	Eric	Chuah	BC Cancer Agency, GCC	echuah@bcgsc.ca		Canada's Michael Smith Genome Sciences Centre, BC Cancer Agency, Vancouver, BC V5Z 4S6		
Ms.	Hye-Jung E.	Chun	BC Cancer Agency, GCC	echun@bcgsc.ca	hchun	Canada's Michael Smith Genome Sciences Centre, BC Cancer Agency, Vancouver, BC V5Z 4S6		
Ms.	Noreen	Dhalla	BC Cancer Agency, GCC	ngirn@bcgsc.ca	nodhalla	Canada's Michael Smith Genome Sciences Centre, BC Cancer Agency, Vancouver, BC V5Z 4S6		
Mr.	Ranabir	Guin	BC Cancer Agency, GCC	rguin@bcgsc.ca		Canada's Michael Smith Genome Sciences Centre, BC Cancer Agency, Vancouver, BC V5Z 4S6		
Dr.	Martin	Hirst	BC Cancer Agency, GCC	mhirst@bcgsc.ca		Canada's Michael Smith Genome Sciences Centre, BC Cancer Agency, Vancouver, BC V5Z 4S6		
Ms.	Carrie	Hirst	BC Cancer Agency, GCC	chirst@bcgsc.ca		Canada's Michael Smith Genome Sciences Centre, BC Cancer Agency, Vancouver, BC V5Z 4S6		
Dr.	Robert A.	Holt	BC Cancer Agency, GCC	rholt@bcgsc.ca		Canada's Michael Smith Genome Sciences Centre, BC Cancer Agency, Vancouver, BC V5Z 4S6		
Dr.	Steven J.M.	Jones	BC Cancer Agency, GCC	sjones@bcgsc.ca		Canada's Michael Smith Genome Sciences Centre, BC Cancer Agency, Vancouver, BC V5Z 4S6		
Ms.	Darlene	Lee	BC Cancer Agency, GCC	dlee@bcgsc.ca		Canada's Michael Smith Genome Sciences Centre, BC Cancer Agency, Vancouver, BC V5Z 4S6		
Ms.	Haiyan I.	Li	BC Cancer Agency, GCC	ili@bcgsc.ca		Canada's Michael Smith Genome Sciences Centre, BC Cancer Agency, Vancouver, BC V5Z 4S6		

Dr.	Marco A.	Marra	BC Cancer Agency, GCC	mmarra@bcgsc.ca	mmarra	Canada's Michael Smith Genome Sciences Centre, BC Cancer Agency, Vancouver, BC V5Z 4S6	
Mr.	Michael	Mayo	BC Cancer Agency, GCC	mmayo@bcgsc.ca		Canada's Michael Smith Genome Sciences Centre, BC Cancer Agency, Vancouver, BC V5Z 4S6	
Dr.	Richard A.	Moore	BC Cancer Agency, GCC	rmoore@bcgsc.ca		Canada's Michael Smith Genome Sciences Centre, BC Cancer Agency, Vancouver, BC V5Z 4S6	
Dr.	Andrew J.	Mungall	BC Cancer Agency, GCC	amungall@bcgsc.ca	amungall	Canada's Michael Smith Genome Sciences Centre, BC Cancer Agency, Vancouver, BC V5Z 4S6	
Dr.	A. Gordon	Robertson	BC Cancer Agency, GCC	grobertson@bcgsc.ca	gorobertson	Canada's Michael Smith Genome Sciences Centre, BC Cancer Agency, Vancouver, BC V5Z 4S6	
Ms.	Jacqueline E.	Schein	BC Cancer Agency, GCC	jschein@bcgsc.ca		Canada's Michael Smith Genome Sciences Centre, BC Cancer Agency, Vancouver, BC V5Z 4S6	
Ms.	Payal	Sipahimalani	BC Cancer Agency, GCC	payals@bcgsc.ca	payals	Canada's Michael Smith Genome Sciences Centre, BC Cancer Agency, Vancouver, BC V5Z 4S6	
Ms.	Angela	Tam	BC Cancer Agency, GCC	atam@bcgsc.ca		Canada's Michael Smith Genome Sciences Centre, BC Cancer Agency, Vancouver, BC V5Z 4S6	
Ms.	Nina	Thiessen	BC Cancer Agency, GCC	nthiessen@bcgsc.ca	nthiessen	Canada's Michael Smith Genome Sciences Centre, BC Cancer Agency, Vancouver, BC V5Z 4S6	
Mr.	Richard J.	Varhol	BC Cancer Agency, GCC	rvarhol@bcgsc.ca		Canada's Michael Smith Genome Sciences Centre, BC Cancer Agency, Vancouver, BC V5Z 4S6	
Mr.	Gordon	Saksena	Broad, GCC; Broad, GDAC	gsaksena@broadinstitute.org		The Eli and Edythe L. Broad Institute of Massachusetts Institute of Technology and Harvard University Cambridge, Massachusetts 02142, USA.	
Mrs.	Juok	Cho	Broad, GDAC	jcho@broadinstitute.org		The Eli and Edythe L. Broad Institute of Massachusetts Institute of Technology and Harvard University Cambridge, Massachusetts 02142, USA.	
Mr.	Daniel	DiCara	Broad, GDAC	dicara@broadinstitute.org		The Eli and Edythe L. Broad Institute of Massachusetts Institute of Technology and Harvard University Cambridge, Massachusetts 02142, USA.	
Mr.	Scott	Frazer	Broad, GDAC	sfrazer@broadinstitute.org		The Eli and Edythe L. Broad Institute of Massachusetts Institute of Technology and Harvard University Cambridge, Massachusetts 02142, USA.	
Dr.	Nils	Gehlenborg	Broad, GDAC	nils@hms.harvard.edu		Center for Biomedical Informatics, Harvard Medical School, Boston, MA 02115, USA	The Eli and Edythe L. Broad Institute of Massachusetts Institute of Technology and Harvard University Cambridge, Massachusetts 02142, USA.
Mr.	David I.	Heiman	Broad, GDAC	dheiman@broadinstitute.org		The Eli and Edythe L. Broad Institute of Massachusetts Institute of Technology and Harvard University Cambridge, Massachusetts 02142, USA.	
Dr.	Jaegil	Kim	Broad, GDAC	jaegil@broadinstitute.org		The Eli and Edythe L. Broad Institute of Massachusetts Institute of Technology and Harvard University Cambridge, Massachusetts 02142, USA.	
Dr.	Michael S.	Lawrence	Broad, GDAC	lawrence@broadinstitute.org		The Eli and Edythe L. Broad Institute of Massachusetts Institute of Technology and Harvard University Cambridge, Massachusetts 02142, USA.	
Mrs.	Pei	Lin	Broad, GDAC	plin@broadinstitute.org		The Eli and Edythe L. Broad Institute of Massachusetts Institute of Technology and Harvard University Cambridge, Massachusetts 02142, USA.	
Dr.	Yingchun	Liu	Broad, GDAC	yingchun@broadinstitute.org		The Eli and Edythe L. Broad Institute of Massachusetts Institute of Technology and Harvard University Cambridge, Massachusetts 02142, USA.	
Mr.	Michael S.	Noble	Broad, GDAC	mnoble@broadinstitute.org		The Eli and Edythe L. Broad Institute of Massachusetts Institute of Technology and Harvard University Cambridge, Massachusetts 02142, USA.	
Mr.	Petar	Stojanov	Broad, GDAC	petar@broadinstitute.org		The Eli and Edythe L. Broad Institute of Massachusetts Institute of Technology and Harvard University Cambridge, Massachusetts 02142, USA.	Department of Medical Oncology, Dana-Farber Cancer Institute, Boston, Massachusetts 02215, USA.
Mr.	Doug	Voet	Broad, GDAC	dvoet@broadinstitute.org		The Eli and Edythe L. Broad Institute of Massachusetts Institute of Technology and Harvard University Cambridge, Massachusetts 02142, USA.	
Dr.	Hailei	Zhang	Broad, GDAC	hailei@broadinstitute.org		The Eli and Edythe L. Broad Institute of Massachusetts Institute of Technology and Harvard University Cambridge, Massachusetts 02142, USA.	

Dr.	Lihua	Zou	Broad, GDAC	lihua@broadinstitute.org	The Eli and Edythe L. Broad Institute of Massachusetts Institute of Technology and Harvard University Cambridge, Massachusetts 02142, USA.		
Dr.	Gad	Getz	Broad, GDAC; Broad, GCC; Broad, GSC	gadgetz@broadinstitute.org	The Eli and Edythe L. Broad Institute of Massachusetts Institute of Technology and Harvard University Cambridge, Massachusetts 02142, USA.	Massachusetts General Hospital Cancer Center and Department of Pathology, Boston, MA 02114, USA	
Dr.	Rameen	Beroukhim	Broad, GCC	rameen@broadinstitute.org	The Eli and Edythe Broad Institute of Harvard and MIT, Cambridge, MA 02142	Departments of Medical Oncology and Cancer Biology, Dana-Farber Cancer Institute, Boston, Massachusetts 02215, USA.	
Dr.	Ami S.	Bhatt	Broad, GCC	asbhatt@broadinstitute.org	The Eli and Edythe L. Broad Institute of Massachusetts Institute of Technology and Harvard University Cambridge, Massachusetts 02142, USA.	Department of Medical Oncology, Dana-Farber Cancer Institute, Boston, Massachusetts 02215, USA	
Dr.	Angela N.	Brooks	Broad, GCC	brooks@broadinstitute.org	The Eli and Edythe L. Broad Institute of Massachusetts Institute of Technology and Harvard University Cambridge, Massachusetts 02142, USA.	Dana-Farber Cancer Institute, Boston, MA 02215	
Dr.	Andrew D.	Cherniack	Broad, GCC	achernia@broadinstitute.prg	The Eli and Edythe Broad Institute of Harvard and MIT, Cambridge, MA 02142		
Mr.	Samuel S	Freeman	Broad, GCC	sfreeman@broadinstitute.org	The Eli and Edythe L. Broad Institute of Massachusetts Institute of Technology and Harvard University Cambridge, Massachusetts 02142, USA.		
Dr.	Stacey B.	Gabriel	Broad, GCC	stacey@broadinstitute.org	The Eli and Edythe Broad Institute of Harvard and MIT, Cambridge, MA 02142		
Ms.	Elena	Helman	Broad, GCC	ehelman@broadinstitute.org	The Eli and Edythe L. Broad Institute of Massachusetts Institute of Technology and Harvard University Cambridge, Massachusetts 02142, USA.	Harvard-MIT Division of Health Sciences & Technology, Cambridge MA	
Dr.	Joonil	Jung	Broad, GCC	joonilj@broadinstitute.org	The Eli and Edythe L. Broad Institute of Massachusetts Institute of Technology and Harvard University Cambridge, Massachusetts 02142, USA.		
Dr.	Matthew	Meyerson	Broad, GCC	matthew_meyerson@dfci.harvard.edu	The Eli and Edythe Broad Institute of Harvard and MIT, Cambridge, MA 02142	Department of Medical Oncology, Dana-Farber Cancer Institute, Boston, Massachusetts 02215, USA.	
Dr.	Akinyemi I	Ojesina	Broad, GCC	ojesina@broadinstitute.org	The Eli and Edythe L. Broad Institute of Massachusetts Institute of Technology and Harvard University Cambridge, Massachusetts 02142, USA.	Department of Medical Oncology, Dana-Farber Cancer Institute, Boston, Massachusetts 02215, USA	
Dr.	Chandra Sekhar	Pedamallu	Broad, GCC	chandra@broadinstitute.org	The Eli and Edythe L. Broad Institute of Massachusetts Institute of Technology and Harvard University Cambridge, Massachusetts 02142, USA.	Department of Medical Oncology, Dana-Farber Cancer Institute, Boston, Massachusetts 02215, USA	
Mr.	Gordon	Saksena	Broad, GCC	gsaksena@broadinstitute.org	The Eli and Edythe Broad Institute of Harvard and MIT, Cambridge, MA 02142		
Mr.	Steven E.	Schumacher	Broad, GCC	schum@broadinstitute.org	The Eli and Edythe Broad Institute of Harvard and MIT, Cambridge, MA 02142	Department of Cancer Biology, Dana-Farber Cancer Institute, Boston, Massachusetts 02215, USA.	
Dr.	Barbara	Tabak	Broad, GCC	barbara.tabak@umassmed.edu	The Eli and Edythe Broad Institute of Harvard and MIT, Cambridge, MA 02142	Department of Cancer Biology, Dana-Farber Cancer Institute, Boston, Massachusetts 02215, USA.	
Mr.	Travis	Zack	Broad, GCC	travis@broadinstitute.org	The Eli and Edythe Broad Institute of Harvard and MIT, Cambridge, MA 02142	Department of Cancer Biology, Dana-Farber Cancer Institute, Boston, Massachusetts 02215, USA.	Biophysics Program, Harvard University, Boston, MA 02115, USA
Dr.	Scott L.	Carter	Broad, GCC; Broad, GDAC	scarter@broadinstitute.org	The Eli and Edythe Broad Institute of Harvard and MIT, Cambridge, MA 02142		
Mr.	Kristian	Cibulskis	Broad, GCC; Broad, GSC	kcibul@broadinstitute.org	The Eli and Edythe L. Broad Institute of Massachusetts Institute of Technology and Harvard University Cambridge, Massachusetts 02142, USA.		
Ms.	Carrie	Sougnez	Broad, GCC; Broad, GSC	carrie@broadinstitute.org	The Eli and Edythe L. Broad Institute of Massachusetts Institute of Technology and Harvard University Cambridge, Massachusetts 02142, USA.		
Dr.	Chip	Stewart	Broad, GDAC	stewart@broadinstitute.org	The Eli and Edythe L. Broad Institute of Massachusetts Institute of Technology and Harvard University Cambridge, Massachusetts 02142, USA.		
Dr.	Eric S.	Lander	Broad, GSC	lander@broadinstitute.org	The Eli and Edythe L. Broad Institute of Massachusetts Institute of Technology and Harvard University Cambridge, Massachusetts 02142, USA.		
Dr	Robert	Burton	DCC	robert.burton@nih.gov	SAIC-Frederick, Inc., 1050 Boyles Street, Frederick, MD 21702 USA		
Dr	Mark A.	Jensen	DCC	mark.jensen@nih.gov	SRA International, Inc., 4300 Fair Lakes Court, Fairfax, VA 22033, USA		
Dr	Ari	Kahn	DCC	ari.kahn.phd@gmail.com	SRA International, Inc., 4300 Fair Lakes Court, Fairfax, VA 22033, USA		
Dr	Todd	Pihl	DCC	todd.pihl@nih.gov	SRA International, Inc., 4300 Fair Lakes Court, Fairfax, VA 22033, USA		
Dr	David	Pot	DCC	potd@mail.nih.gov	SRA International, Inc., 4300 Fair Lakes Court, Fairfax, VA 22033, USA		
Dr	Yunhu	Wan	DCC	wany@mail.nih.gov	SRA International, Inc., 4300 Fair Lakes Court, Fairfax, VA 22033, USA		

Dr.	Christopher A.	Bristow	Harvard/BWH/MDACC, GCC	CABristow@mdanderson.org	Institute for Applied Cancer Science, Department of Genomic Medicine, University of Texas MD Anderson Cancer Center, Houston, TX 77030		
Dr.	Angela	Hadjipanayis	Harvard/BWH/MDACC, GCC	ahadjipanayis@partners.org	Department of Genetics, Harvard Medical School, Boston, MA 02115 USA		
Ms.	Psalm	Haseley	Harvard/BWH/MDACC, GCC	psm3426@gmail.com	The Center for Biomedical Informatics, Harvard Medical School, Boston, MA 02115, USA		
Dr.	Raju	Kucherlapati	Harvard/BWH/MDACC, GCC	rkucherlapati@partners.org	Harvard Medical School-Partners HealthCare Center for Genetics and Genomics, Boston, Massachusetts 02115, USA.		
Dr.	Semin	Lee	Harvard/BWH/MDACC, GCC	Semin_Lee@hms.harvard.edu	The Center for Biomedical Informatics, Harvard Medical School, Boston, MA 02115, USA		
Dr.	Eunjung	Lee	Harvard/BWH/MDACC, GCC	ejalice.lee@gmail.com	The Center for Biomedical Informatics, Harvard Medical School, Boston, MA 02115, USA		
Mr.	Lovelace J.	Luquette	Harvard/BWH/MDACC, GCC	Lovelace_Luquette@hms.harvard.edu	The Center for Biomedical Informatics, Harvard Medical School, Boston, MA 02115, USA		
Mr.	Harshad S.	Mahadeshwar	Harvard/BWH/MDACC, GCC	HSMahadeshwar@mdanderson.org	Institute for Applied Cancer Science, Department of Genomic Medicine, University of Texas MD Anderson Cancer Center, Houston, TX 77030		
Dr.	Angeliki	Pantazi	Harvard/BWH/MDACC, GCC	apantazi@partners.org	Department of Genetics, Harvard Medical School, Boston, MA 02115 USA		
Dr.	Michael	Parfenov	Harvard/BWH/MDACC, GCC	parfenov@genetics.med.harvard.edu	Department of Genetics, Harvard Medical School, Boston, MA 02115 USA		
Dr.	Peter J.	Park	Harvard/BWH/MDACC, GCC	peter_park@hms.harvard.edu	The Center for Biomedical Informatics, Harvard Medical School, Boston, MA 02115, USA	Division of Genetics, Brigham and Women's Hospital, Boston, Massachusetts 02115, USA	Informatics Program, Children's Hospital, Boston, Massachusetts 02115, USA
Dr.	Alexei	Protopopov	Harvard/BWH/MDACC, GCC	aprotopopov@mdanderson.org	Institute for Applied Cancer Science, Department of Genomic Medicine, The University of Texas MD Anderson Cancer Center, Houston, TX 77030 USA		
Ms.	Xiaojia	Ren	Harvard/BWH/MDACC, GCC	Xiaojia_Ren@dfci.harvard.edu	Department of Genetics, Harvard Medical School, Boston, MA 02115 USA		
Ms.	Netty	Santoso	Harvard/BWH/MDACC, GCC	nsantoso@partners.org	Department of Genetics, Harvard Medical School, Boston, MA 02115 USA		
Dr.	Jonathan	Seidman	Harvard/BWH/MDACC, GCC	seidman@genetics.med.harvard.edu	Department of Genetics, Harvard Medical School, Boston, Massachusetts 02115, USA.		
Mr.	Sahil	Seth	Harvard/BWH/MDACC, GCC	SSeth@mdanderson.org	Institute for Applied Cancer Science, Department of Genomic Medicine, University of Texas MD Anderson Cancer Center, Houston, TX 77030		
Dr.	Xingzhi	Song	Harvard/BWH/MDACC, GCC	XSong3@mdanderson.org	Institute for Applied Cancer Science, Department of Genomic Medicine, University of Texas MD Anderson Cancer Center, Houston, TX 77030		
Dr.	Jiabin	Tang	Harvard/BWH/MDACC, GCC	JTang3@mdanderson.org	Institute for Applied Cancer Science, Department of Genomic Medicine, University of Texas MD Anderson Cancer Center, Houston, TX 77030		
Dr.	Ruibin	Xi	Harvard/BWH/MDACC, GCC	ruibinxi@gmail.com	The Center for Biomedical Informatics, Harvard Medical School, Boston, MA 02115, USA	School of Mathematical Sciences and Center for Statistical Science, Peking University, Beijing, China	
Dr.	Andrew W.	Xu	Harvard/BWH/MDACC, GCC	wxu7@partners.org	The Center for Biomedical Informatics, Harvard Medical School, Boston, MA 02115, USA		
Dr.	Lixing	Yang	Harvard/BWH/MDACC, GCC	lixing_yang@hms.harvard.edu	The Center for Biomedical Informatics, Harvard Medical School, Boston, MA 02115, USA		
Mr.	Dong	Zeng	Harvard/BWH/MDACC, GCC	dzeng@mdanderson.org	Institute for Applied Cancer Science, Department of Genomic Medicine, University of Texas MD Anderson Cancer Center, Houston, TX 77030		
Dr.	Lynda	Chin	Harvard/BWH/MDACC, GCC; Broad, GDAC	lchin@mdanderson.org	Institute for Applied Cancer Science, Department of Genomic Medicine, The University of Texas MD Anderson Cancer Center, Houston, TX 77030 USA	The Eli and Edythe L. Broad Institute of Massachusetts Institute Of Technology and Harvard University Cambridge, MA 02142	
Dr.	Jianhua	Zhang	Harvard/BWH/MDACC, GCC; Broad, GDAC	jzhang22@mdanderson.org	Institute for Applied Cancer Science, Department of Genomic Medicine, The University of Texas MD Anderson Cancer Center, Houston, TX 77030 USA		
Dr.	Brady	Bernard	ISB/MDACC, GDAC	brady.bernard@systemsbiology.org	Institute for Systems BIology, Seattle, WA 98109		
	Ryan	Bressler	ISB/MDACC, GDAC	ryan.bressler@systemsbiology.org	Institute for Systems BIology, Seattle, WA 98109		
	Andrea	Eakin	ISB/MDACC, GDAC	andrea.eakin@systemsbiology.org	Institute for Systems BIology, Seattle, WA 98109		
Dr.	Lisa	Iype	ISB/MDACC, GDAC	liype@systemsbiology.org	Institute for Systems BIology, Seattle, WA 98109		
Dr.	Theo	Knijnenburg	ISB/MDACC, GDAC	theo.knijnenburg@systemsbiology.org	Institute for Systems BIology, Seattle, WA 98109		
	Roger	Kramer	ISB/MDACC, GDAC	roger.kramer@systemsbiology.org	Institute for Systems BIology, Seattle, WA 98109		
	Richard	Kreisberg	ISB/MDACC, GDAC	dick.kreisberg@systemsbiology.org	Institute for Systems BIology, Seattle, WA 98109		
	Kalle	Leinonen	ISB/MDACC, GDAC	kalle.leinonen@systemsbiology.org	Institute for Systems Biology, Seattle, WA, 98109		
	Jake	Lin	ISB/MDACC, GDAC	jake.lin@systemsbiology.org	Institute for Systems BIology, Seattle, WA 98109		
Dr.	Yuexin	Liu	ISB/MDACC, GDAC	YLiu8@mdanderson.org	MD Anderson Cancer Center, Houston, TX 77030		

	Michael	Miller	ISB/MDACC, GDAC	michael.miller@systemsbiology.org		Institute for Systems BIology, Seattle, WA 98109	
Dr.	Sheila M.	Reynolds	ISB/MDACC, GDAC	sheila.reynolds@systemsbiology.org		Institute for Systems BIology, Seattle, WA 98109	
	Hector	Rovira	ISB/MDACC, GDAC	hector.rovira@systemsbiology.org		Institute for Systems BIology, Seattle, WA 98109	
Dr.	Ilya	Shmulevich	ISB/MDACC, GDAC	ilya.shmulevich@systemsbiology.org		Institute for Systems BIology, Seattle, WA 98109	
Dr.	Vesteinn	Thorsson	ISB/MDACC, GDAC	vesteinn.thorsson@systemsbiology.org		Institute for Systems BIology, Seattle, WA 98109	
Dr.	Da	Yang	ISB/MDACC, GDAC	dyang3@mdanderson.org		MD Anderson Cancer Center, Houston, TX 77030	
Dr.	Wei	Zhang	ISB/MDACC, GDAC	wzhang@mdanderson.org		MD Anderson Cancer Center, Houston, TX 77030	
Mr.	Samirkumar	Amin	Broad, GDAC	samin11@mdanderson.org		Department of Genomic Medicine, The University of Texas MD Anderson Cancer Center, Houston, TX 77030 USA	
Dr.	Chang-Jiun	Wu	Broad, GDAC	cwu7@mdanderson.org		Institute for Applied Cancer Science, Department of Genomic Medicine, The University of Texas MD Anderson Cancer Center, Houston, TX 77030 USA	
Dr.	Chia-Chin	Wu	Broad, GDAC	cwu9@mdanderson.org		Institute for Applied Cancer Science, Department of Genomic Medicine, The University of Texas MD Anderson Cancer Center, Houston, TX 77030 USA	
Dr.	Rehan	Akbani	MDACC, GDAC	rakbani@mdanderson.org	rakbani	UT MD Anderson Cancer Center, Bioinformatics and Computational Biology, 1400 Pressler Street, Unit 1410, Houston, TX 77030	
Dr.	Kenneth	Aldape	MDACC, GDAC	kaldape@mdanderson.org		Department of Pathology, University of Texas MD Anderson Cancer Center, Houston, TX 77030	
Dr.	Keith A	Baggerly	MDACC, GDAC	kabagger@mdanderson.org		UT MD Anderson Cancer Center, Bioinformatics and Computational Biology, 1400 Pressler Street, Unit 1410, Houston, TX 77030	
Dr.	Bradley	Broom	MDACC, GDAC	bmbroom@mdanderson.org		UT MD Anderson Cancer Center, Bioinformatics and Computational Biology, 1400 Pressler Street, Unit 1410, Houston, TX 77030	
	Tod D	Casasent	MDACC, GDAC	TDCasasent@mdanderson.org		UT MD Anderson Cancer Center, Bioinformatics and Computational Biology, 1400 Pressler Street, Unit 1410, Houston, TX 77030	
	James	Cleland	MDACC, GDAC	JACleland@mdanderson.org		UT MD Anderson Cancer Center, Bioinformatics and Computational Biology, 1400 Pressler Street, Unit 1410, Houston, TX 77030	In Silico Solutions, 11781 Lee Jackson Memorial Hwy Ste 320. Fairfax, VA 22033
Dr.	Chad	Creighton	MDACC, GDAC	creighto@bcm.edu		Baylor College of Medicine	
	Deepti	Dodda	MDACC, GDAC	DDodda@mdanderson.org		UT MD Anderson Cancer Center, Bioinformatics and Computational Biology, 1400 Pressler Street, Unit 1410, Houston, TX 77030	
Dr.	Mary	Edgerton	MDACC, GDAC	medgerton@mdanderson.org		UT MD Anderson Cancer Center, Dept of Pathology	
Dr.	Leng	Han	MDACC, GDAC	lhan1@mdanderson.org		UT MD Anderson Cancer Center, Bioinformatics and Computational Biology, 1400 Pressler Street, Unit 1410, Houston, TX 77030	
	Shelley M	Herbrich	MDACC, GDAC	SMHerbrich@mdanderson.org		UT MD Anderson Cancer Center, Bioinformatics and Computational Biology, 1400 Pressler Street, Unit 1410, Houston, TX 77030	
	Zhenlin	Ju	MDACC, GDAC	zju@mdanderson.org		UT MD Anderson Cancer Center, Bioinformatics and Computational Biology, 1400 Pressler Street, Unit 1410, Houston, TX 77030	
Dr.	Hoon	Kim	MDACC, GDAC	hkim6@mdanderson.org		UT MD Anderson Cancer Center, Bioinformatics and Computational Biology, 1400 Pressler Street, Unit 1410, Houston, TX 77030	
Dr.	Seth	Lerner	MDACC, GDAC	slerner@bcm.edu		Baylor College of Medicine, Dept of Urology	
	Jun	Li	MDACC, GDAC	jli14@mdanderson.org		UT MD Anderson Cancer Center, Bioinformatics and Computational Biology, 1400 Pressler Street, Unit 1410, Houston, TX 77030	
Dr.	Han	Liang	MDACC, GDAC	hliang1@mdanderson.org		UT MD Anderson Cancer Center, Bioinformatics and Computational Biology, 1400 Pressler Street, Unit 1410, Houston, TX 77030	
	Wenbin	Liu	MDACC, GDAC	wliu@mdanderson.org			
Dr.	Philip L.	Lorenzi	MDACC, GDAC	pllorenzi@mdanderson.org		UT MD Anderson Cancer Center, Bioinformatics and Computational Biology, 1400 Pressler Street, Unit 1410, Houston, TX 77030	

Dr.	Yiling	Lu	MDACC, GDAC	yilinglu@mdanderson.org	UT MD Anderson Cancer Center, Systems Biology Dept, 1515 Holcombe Blvd, Houston, TX, 77030	
	James	Melott	MDACC, GDAC	JMMelott@mdanderson.org	UT MD Anderson Cancer Center, Bioinformatics and Computational Biology, 1400 Pressler Street, Unit 1410, Houston, TX 77030	
Dr.	Gordon B	Mills	MDACC, GDAC	gmills@mdanderson.org	UT MD Anderson Cancer Center, Systems Biology Dept, 1515 Holcombe Blvd, Houston, TX, 77030	
	Lam	Nguyen	MDACC, GDAC	am_Nguyen@sra.com	UT MD Anderson Cancer Center, Bioinformatics and Computational Biology, 1400 Pressler Street, Unit 1410, Houston, TX 77030	In Silico Solutions, 11781 Lee Jackson Memorial Hwy Ste 320. Fairfax, VA 22033
Dr.	Xiaoping	Su	MDACC, GDAC	XSu1@mdanderson.org	UT MD Anderson Cancer Center, Bioinformatics and Computational Biology, 1400 Pressler Street, Unit 1410, Houston, TX 77030	
Dr.	Roeland	Verhaak	MDACC, GDAC	rverhaak@mdanderson.org	UT MD Anderson Cancer Center, Bioinformatics and Computational Biology, 1400 Pressler Street, Unit 1410, Houston, TX 77030	
Dr.	Wenyi	Wang	MDACC, GDAC	WWang7@mdanderson.org	UT MD Anderson Cancer Center, Bioinformatics and Computational Biology, 1400 Pressler Street, Unit 1410, Houston, TX 77030	
Dr.	John N.	Weinstein	MDACC, GDAC	jweinstei@mdanderson.org	UT MD Anderson Cancer Center, Bioinformatics and Computational Biology; Systems Biology, 1400 Pressler Street, Unit 1410, Houston, TX 77030	
	Andrew	Wong	MDACC, GDAC	awong@insilico.us.com	UT MD Anderson Cancer Center, Bioinformatics and Computational Biology, 1400 Pressler Street, Unit 1410, Houston, TX 77030	In Silico Solutions, 11781 Lee Jackson Memorial Hwy Ste 320. Fairfax, VA 22033
	Yang	Yang	MDACC, GDAC	Yang.Yang@uth.tmc.edu	Division of Biostatistics, The University of Texas Health Science Center at Houston, School of Public Health, Houston TX 77030, USA	UT MD Anderson Cancer Center, Bioinformatics and Computational Biology, 1400 Pressler Street, Unit 1410, Houston, TX 77030
Dr.	Jun	Yao	MDACC, GDAC	JYao1@mdanderson.org	UT MD Anderson Cancer Center, Neuro-Oncology Dept, 1515 Holcombe Blvd, Houston, TX, 77030	
	Rong	Yao	MDACC, GDAC	RYao@mdanderson.org	UT MD Anderson Cancer Center, Bioinformatics and Computational Biology; Systems Biology, 1400 Pressler Street, Unit 1410, Houston, TX 77030	
Dr.	Kosuke	Yoshihara	MDACC, GDAC	kyoshihara@mdanderson.org	UT MD Anderson Cancer Center, Bioinformatics and Computational Biology, 1400 Pressler Street, Unit 1410, Houston, TX 77030	
	Yuan	Yuan	MDACC, GDAC	yuanyuan0116@gmail.com	Graduate Program in Structural and Computational Biology and Molecular Biophysics, Baylor College of Medicine, Houston, TX, USA	UT MD Anderson Cancer Center, Bioinformatics and Computational Biology, 1400 Pressler Street, Unit 1410, Houston, TX 77030
Dr.	Alfred K.	Yung	MDACC, GDAC	wyung@mdanderson.org	UT MD Anderson Cancer Center, Neuro-Oncology Dept, 1515 Holcombe Blvd, Houston, TX, 77030	
Dr.	Nianxiang	Zhang	MDACC, GDAC	nxzhang@hotmail.com	UT MD Anderson Cancer Center, Bioinformatics and Computational Biology, 1400 Pressler Street, Unit 1410, Houston, TX 77030	
Dr.	Siyuan	Zheng	MDACC, GDAC	szheng2@mdanderson.org	UT MD Anderson Cancer Center, Bioinformatics and Computational Biology, 1400 Pressler Street, Unit 1410, Houston, TX 77030	
Dr.	Michael	Ryan	MDACC, GDAC	mryan@insilico.us.com	UT MD Anderson Cancer Center, Systems Biology Dept, 1515 Holcombe Blvd, Houston, TX, 77030	In Silico Solutions, 11781 Lee Jackson Memorial Hwy Ste 320. Fairfax, VA 22033
Dr.	David W	Kane	MDACC, GDAC	David_Kane@sra.com	UT MD Anderson Cancer Center, Bioinformatics and Computational Biology, 1400 Pressler Street, Unit 1410, Houston, TX 77030	SRA International
	B. Arman	Aksoy	MSKCC, GDAC	arman@cbio.mskcc.org	Computational Biology Center, Memorial Sloan-Kettering Cancer Center, New York, New York 10065, USA	
Dr.	Giovanni	Ciriello	MSKCC, GDAC	ciriello@cbio.mskcc.org	Computational Biology Center, Memorial Sloan-Kettering Cancer Center, New York, New York 10065, USA	
	Gideon	Dresdner	MSKCC, GDAC	dresdner@cbio.mskcc.org	Computational Biology Center, Memorial Sloan-Kettering Cancer Center, New York, New York 10065, USA	
Dr.	Jianjiong	Gao	MSKCC, GDAC	jgao@cbio.mskcc.org	Computational Biology Center, Memorial Sloan-Kettering Cancer Center, New York, New York 10065, USA	
	Benjamin	Gross	MSKCC, GDAC	grossb@cbio.mskcc.org	Computational Biology Center, Memorial Sloan-Kettering Cancer Center, New York, New York 10065, USA	
Dr.	Anders	Jacobsen	MSKCC, GDAC	jacobsen@cbio.mskcc.org	Computational Biology Center, Memorial Sloan-Kettering Cancer Center, New York, New York 10065, USA	

Mr.	Andre	Kahles	MSKCC, GDAC	akahles@cbio.mskcc.org	Computational Biology Center, Memorial Sloan-Kettering Cancer Center, New York, New York 10065, USA	
Dr.	Marc	Ladanyi	MSKCC, GDAC	ladanyim@mskcc.org	Department of Pathology and Human Oncology & Pathogenesis Program, Memorial Sloan-Kettering Cancer Center, New York, New York 10065, USA.	
Dr.	William	Lee	MSKCC, GDAC	leew1@cbio.mskcc.org	Computational Biology Center, Memorial Sloan-Kettering Cancer Center, New York, New York 10065, USA	
Dr.	Kjong-Van	Lehmann	MSKCC, GDAC	lehmann@cbio.mskcc.org	Computational Biology Center, Memorial Sloan-Kettering Cancer Center, New York, New York 10065, USA	
Dr.	Martin L.	Miller	MSKCC, GDAC	miller@cbio.mskcc.org	Computational Biology Center, Memorial Sloan-Kettering Cancer Center, New York, New York 10065, USA	
	Ricardo	Ramirez	MSKCC, GDAC	rir2010@cbio.mskcc.org	Computational Biology Center, Memorial Sloan-Kettering Cancer Center, New York, New York 10065, USA	
Dr.	Gunnar	Rätsch	MSKCC, GDAC	raetsch@cbio.mskcc.org	Computational Biology Center, Memorial Sloan-Kettering Cancer Center, New York, New York 10065, USA	
Dr.	Boris	Reva	MSKCC, GDAC	borisr@mskcc.org	Computational Biology Center, Memorial Sloan-Kettering Cancer Center, New York, New York 10065, USA	
Dr.	Chris	Sander	MSKCC, GDAC	sanderc@mskcc.org	Computational Biology Center, Memorial Sloan-Kettering Cancer Center, New York, New York 10065, USA	
Dr.	Nikolaus	Schultz	MSKCC, GDAC	schultz@cbio.mskcc.org	Computational Biology Center, Memorial Sloan-Kettering Cancer Center, New York, New York 10065, USA	
Dr.	Yasin	Senbabaoglu	MSKCC, GDAC	yasin@cbio.mskcc.org	Computational Biology Center, Memorial Sloan-Kettering Cancer Center, New York, New York 10065, USA	
Dr.	Ronglai	Shen	MSKCC, GDAC	shenr@mskcc.org	Department of Epidemiology and Biostatistics, Memorial Sloan-Kettering Cancer Center, New York, New York 10065, USA	
Dr.	Rileen	Sinha	MSKCC, GDAC	rileen@cbio.mskcc.org	Computational Biology Center, Memorial Sloan-Kettering Cancer Center, New York, New York 10065, USA	
	S. Onur	Sumer	MSKCC, GDAC	onur@cbio.mskcc.org	Computational Biology Center, Memorial Sloan-Kettering Cancer Center, New York, New York 10065, USA	
	Yichao	Sun	MSKCC, GDAC	suny1@mskcc.org	Computational Biology Center, Memorial Sloan-Kettering Cancer Center, New York, New York 10065, USA	
Dr.	Barry S.	Taylor	MSKCC, GDAC	TaylorBS@cc.ucsf.edu	Departments of Epidemiology and Biostatistics, Medicine, and the Helen Diller Family Comprehensive Cancer Center, University of California, San Francisco, 94158, USA	
Dr.	Nils	Weinhold	MSKCC, GDAC	weinhold@cbio.mskcc.org	Computational Biology Center, Memorial Sloan-Kettering Cancer Center, New York, New York 10065, USA	
Dr.	Douglas A.	Levine	MSKCC, TSS	levine2@MSKCC.ORG	Gynecology Service, Department of Surgery, Memorial Sloan-Kettering Cancer Center, New York, NY 10065	
Mr.	Aaron D.	Black	NCH, BCR	Aaron.Black@nationwidechildrens.org	The Research Institute at Nationwide Children's Hospital, Columbus, OH 43205	
Mr.	Jay	Bowen	NCH, BCR	Jay.Bowen@nationwidechildrens.org	The Research Institute at Nationwide Children's Hospital, Columbus, OH 43205	
Ms.	Jessica	Frick	NCH, BCR	Jessica.Frick@nationwidechildrens.org	The Research Institute at Nationwide Children's Hospital, Columbus, OH 43205	
Dr.	Julie M.	Gastier-Foster	NCH, BCR	Julie.Gastier-Foster@nationwidechildrens.org	The Research Institute at Nationwide Children's Hospital, Columbus, OH 43205	The Ohio State University, Columbus, OH 43210, USA
Ms.	Hollie A.	Harper	NCH, BCR	Hollie.Harper@nationwidechildrens.org	The Research Institute at Nationwide Children's Hospital, Columbus, OH 43205	
Ms.	Carmen	Helsel	NCH, BCR	Carmen.Helsel@nationwidechildrens.org	The Research Institute at Nationwide Children's Hospital, Columbus, OH 43205	
Ms.	Kristen M.	Leraas	NCH, BCR	Kristen.Leraas@nationwidechildrens.org	The Research Institute at Nationwide Children's Hospital, Columbus, OH 43205	
Ms.	Tara M.	Lichtenberg	NCH, BCR	Tara.Lichtenberg@nationwidechildrens.org	The Research Institute at Nationwide Children's Hospital, Columbus, OH 43205	
Ms.	Cynthia	McAllister	NCH, BCR	Cynthia.McAllister@nationwidechildrens.org	The Research Institute at Nationwide Children's Hospital, Columbus, OH 43205	
Dr.	Nilsa C.	Ramirez	NCH, BCR	Nilsa.Ramirez@nationwidechildrens.org	The Research Institute at Nationwide Children's Hospital, Columbus, OH 43205	The Ohio State University, Columbus, OH 43210, USA
Ms.	Samantha	Sharpe	NCH, BCR	Samantha.Sharpe@nationwidechildrens.org	The Research Institute at Nationwide Children's Hospital, Columbus, OH 43205	
Ms.	Lisa	Wise	NCH, BCR	Lisa.Wise@nationwidechildrens.org	The Research Institute at Nationwide Children's Hospital, Columbus, OH 43205	
Dr.	Erik	Zmuda	NCH, BCR	Erik.Zmuda@nationwidechildrens.org	The Research Institute at Nationwide Children's Hospital, Columbus, OH 43205	
Dr.	Stephen	Chanock	NCI/NHGRI Project Team	Stephen.Chanock@nih.gov	National Cancer Institute, NIH, Bethesda, MD 20892	
Dr.	Tanja	Davidsen	NCI/NHGRI Project Team	tanja.davidsen@nih.gov	National Cancer Institute, NIH, Bethesda, MD 20892	
Mr.	John A.	Demchok	NCI/NHGRI Project Team	john.demchok@nih.gov	National Cancer Institute, NIH, Bethesda, MD 20892	

Dr.	Greg	Eley	NCI/NHGRI Project Team	geley@scimentis.com		Scimentis Statham, GA 30666	
Ms.	Ina	Felau	NCI/NHGRI Project Team	ina.felau@nih.gov		National Cancer Institute, NIH, Bethesda, MD 20892	
Dr.	Brad	Ozenberger	NCI/NHGRI Project Team	bozenberger@mail.nih.gov		National Human Genome Research Institute, NIH, Bethesda, MD 20892	
Ms.	Margi	Sheth	NCI/NHGRI Project Team	margi.sheth@nih.gov		National Cancer Institute, NIH, Bethesda, MD 20892	
Dr.	Heidi	Sofia	NCI/NHGRI Project Team	Heidi.Sofia@nih.gov		National Human Genome Research Institute, NIH, Bethesda, MD 20892	
Dr.	Louis	Staudt	NCI/NHGRI Project Team	Louis.Staudt@nih.gov		National Cancer Institute, NIH, Bethesda, MD 20892	
Dr.	Roy	Tarnuzzer	NCI/NHGRI Project Team	tarnuzzerrw@mail.nih.gov		National Cancer Institute, NIH, Bethesda, MD 20892	
Dr.	Zhining	Wang	NCI/NHGRI Project Team	zhining.wang@nih.gov		National Cancer Institute, NIH, Bethesda, MD 20892	
Dr.	Liming	Yang	NCI/NHGRI Project Team	lyang@mail.nih.gov		National Cancer Institute, NIH, Bethesda, MD 20892	
Ms.	Jiashan	Zhang	NCI/NHGRI Project Team	zhangjul@mail.nih.gov		National Cancer Institute, NIH, Bethesda, MD 20892	
Dr.	Suzanne	Fei	OHSU, GDAC	feis@ohsu.edu		Oregon Health & Science University, Portland, OR 97239	
Dr.	Paul	Spellman	OHSU, GDAC	spellmap@ohsu.edu		Oregon Health & Science University, Portland, OR 97240	
Dr.	Christopher	Benz	UCSC/Buck, GDAC	cbenz@buckinstitute.org		Buck Institute for Research on Aging, Novato, CA 94945	
Mr.	Daniel	Carlin	UCSC/Buck, GDAC	decarlin@ucsc.edu		Univ. Calif. Santa Cruz, 1156 High St., Santa Cruz, CA 95064	
Dr.	Melisssa	Cline	UCSC/Buck, GDAC	cline@soe.ucsc.edu		Univ. Calif. Santa Cruz, 1156 High St., Santa Cruz, CA 95064	
Mr.	Brian	Craft	UCSC/Buck, GDAC	craft@soe.ucsc.edu		Univ. Calif. Santa Cruz, 1156 High St., Santa Cruz, CA 95064	
Dr.	Kyle	Ellrott	UCSC/Buck, GDAC	kellrott@soe.ucsc.edu		Univ. Calif. Santa Cruz, 1156 High St., Santa Cruz, CA 95064	
Ms.	Mary	Goldman	UCSC/Buck, GDAC	mary@soe.ucsc.edu		Univ. Calif. Santa Cruz, 1156 High St., Santa Cruz, CA 95064	
Dr.	David	Haussler	UCSC/Buck, GDAC	haussler@soe.ucsc.edu		Univ. Calif. Santa Cruz, 1156 High St., Santa Cruz, CA 95064	Howard Hughes Medical Institute, Univ. Calif. Santa Cruz, 1156 High St., Santa Cruz, CA 95064
Mr.	Singer	Ma	UCSC/Buck, GDAC	singer@soe.ucsc.edu		Univ. Calif. Santa Cruz, 1156 High St., Santa Cruz, CA 95064	
	Sam	Ng	UCSC/Buck, GDAC	sng@soe.ucsc.edu		Univ. Calif. Santa Cruz, 1156 High St., Santa Cruz, CA 95064	
	Evan	Paull	UCSC/Buck, GDAC	epaull@soe.ucsc.edu	evpaull	Univ. Calif. Santa Cruz, 1156 High St., Santa Cruz, CA 95064	
Ms.	Amie	Radenbaugh	UCSC/Buck, GDAC	aradenba@soe.ucsc.edu		Univ. Calif. Santa Cruz, 1156 High St., Santa Cruz, CA 95064	
Dr.	Sofie	Salama	UCSC/Buck, GDAC	ssalama@soe.ucsc.edu		Univ. Calif. Santa Cruz, 1156 High St., Santa Cruz, CA 95064	Howard Hughes Medical Institute, Univ. Calif. Santa Cruz, 1156 High St., Santa Cruz, CA 95064
Dr.	Artem	Sokolov	UCSC/Buck, GDAC	sokolov@soe.ucsc.edu		Univ. Calif. Santa Cruz, 1156 High St., Santa Cruz, CA 95064	
Dr.	Joshua M.	Stuart	UCSC/Buck, GDAC	jstuart@ucsc.edu		Univ. Calif. Santa Cruz, 1156 High St., Santa Cruz, CA 95064	
Ms.	Teresa	Swatloski	UCSC/Buck, GDAC	swat@soe.ucsc.edu		Univ. Calif. Santa Cruz, 1156 High St., Santa Cruz, CA 95064	
	Vladislav	Uzunangelov	UCSC/Buck, GDAC	uzunangelov@soe.ucsc.edu		Univ. Calif. Santa Cruz, 1156 High St., Santa Cruz, CA 95064	
Dr.	Peter	Waltman	UCSC/Buck, GDAC	pwaltman@ucsc.edu		Univ. Calif. Santa Cruz, 1156 High St., Santa Cruz, CA 95064	
Dr.	Christina	Yau	UCSC/Buck, GDAC	cyau@buckinstitute.org		Buck Institute for Research on Aging, Novato, CA 94945	
Dr.	Jing	Zhu	UCSC/Buck, GDAC	jzhu@soe.ucsc.edu		Univ. Calif. Santa Cruz, 1156 High St., Santa Cruz, CA 95064	
Dr.	J.Todd	Auman	UNC, GCC	jtauman@email.unc.edu	jtauman	Eshelman School of Pharmacy, University of North Carolina at Chapel Hill, Chapel Hill, NC 27599 USA	
Mr.	Saianand	Balu	UNC, GCC	sai@unc.edu		Lineberger Comprehensive Cancer Center, University of North Carolina at Chapel Hill, Chapel Hill, NC 27599 USA	
Ms.	Elizabeth	Buda	UNC, GCC	ebuda@email.unc.edu		Carolina Center for Genome Sciences, University of North Carolina at Chapel Hill, Chapel Hill, NC 27599 USA	
Mr.	Cheng	Fan	UNC, GCC	cfan2004@gmail.com		Lineberger Comprehensive Cancer Center, University of North Carolina at Chapel Hill, Chapel Hill, NC 27599 USA	
Dr.	Katherine A.	Hoadley	UNC, GCC	hoadley@med.unc.edu		Lineberger Comprehensive Cancer Center, University of North Carolina at Chapel Hill, Chapel Hill, NC 27599 USA	
Dr.	Corbin D.	Jones	UNC, GCC	cdjones@email.unc.edu		Department of Biology, University of North Carolina at Chapel Hill, Chapel Hill, NC 27599 USA	Carolina Center for Genome Sciences, University of North Carolina at Chapel Hill, Chapel Hill, NC 27599 USA
Dr.	Shaowu	Meng	UNC, GCC	shaowu_meng@med.unc.edu		Lineberger Comprehensive Cancer Center, University of North Carolina at Chapel Hill, Chapel Hill, NC 27599 USA	
Dr.	Piotr A.	Mieczkowski	UNC, GCC	Piotr_Mieczkowski@med.unc.edu		Department of Genetics, University of North Carolina at Chapel Hill, Chapel Hill, NC 27599 USA	

Dr.	Joel S.	Parker	UNC, GCC	parkerjs@email.unc.edu		Department of Genetics, University of North Carolina at Chapel Hill, Chapel Hill, NC 27599 USA	Lineberger Comprehensive Cancer Center, University of North Carolina at Chapel Hill, Chapel Hill, NC 27599 USA	
Dr.	Charles M.	Perou	UNC, GCC	cperou@med.unc.edu	chperou	Department of Genetics, University of North Carolina at Chapel Hill, Chapel Hill, NC 27599 USA	Department of Pathology and Laboratory Medicine, University of North Carolina at Chapel Hill, Chapel Hill, Chapel Hill, NC 27599 USA	Lineberger Comprehensive Cancer Center, UNC Chapel Hill, 450 West Drive. Chapel Hill, NC 27599
Dr.	Jeffrey	Roach	UNC, GCC	jeff_roach@unc.edu		Research Computing Center, University of North Carolina at Chapel Hill, Chapel Hill, NC 27599 USA		
Dr.	Yan	Shi	UNC, GCC	yan_shi@med.unc.edu		Lineberger Comprehensive Cancer Center, University of North Carolina at Chapel Hill, Chapel Hill, NC 27599 USA		
Ms.	Grace O.	Silva	UNC, GCC	silvag@email.unc.edu		Department of Genetics, University of North Carolina at Chapel Hill, Chapel Hill, NC 27599 USA	Lineberger Comprehensive Cancer Center, University of North Carolina at Chapel Hill, Chapel Hill, NC 27599 USA	
Ms.	Donghui	Tan	UNC, GCC	donghui_tan@med.unc.edu		Department of Genetics, University of North Carolina at Chapel Hill, Chapel Hill, NC 27599 USA		
Ms.	Umadevi	Veluvolu	UNC, GCC	umadevi_veluvolu@med.unc.edu		Department of Genetics, University of North Carolina at Chapel Hill, Chapel Hill, NC 27599 USA		
Dr.	Scot	Waring	UNC, GCC	swaring@gmail.com		Carolina Center for Genome Sciences, University of North Carolina at Chapel Hill, Chapel Hill, NC 27599 USA	Lineberger Comprehensive Cancer Center, University of North Carolina at Chapel Hill, Chapel Hill, NC 27599 USA	
Dr.	Matthew D.	Wilkerson	UNC, GCC	matthew_wilkerson@med.unc.edu		Department of Genetics, University of North Carolina at Chapel Hill, Chapel Hill, NC 27599 USA		
Mr.	Junyuan	Wu	UNC, GCC	georgewu@med.unc.edu		Lineberger Comprehensive Cancer Center, University of North Carolina at Chapel Hill, Chapel Hill, NC 27599 USA		
Ms.	Wei	Zhao	UNC, GCC	zhaow@email.unc.edu		Department of Genetics, University of North Carolina at Chapel Hill, Chapel Hill, NC 27599 USA	Lineberger Comprehensive Cancer Center, University of North Carolina at Chapel Hill, Chapel Hill, NC 27599 USA	
Mr.	Tom	Bodenheimer	UNC, GDAC	bodenhei@email.unc.edu		Lineberger Comprehensive Cancer Center, University of North Carolina at Chapel Hill, Chapel Hill, NC 27599 USA		
Dr.	D. Neil	Hayes	UNC, GDAC	hayes@med.unc.edu		Department of Internal Medicine, Division of Medical Oncology, University of North Carolina at Chapel Hill, Chapel Hill, NC 27599 USA	Lineberger Comprehensive Cancer Center, University of North Carolina at Chapel Hill, Chapel Hill, NC 27599 USA	
Dr.	D. Neil	Hayes	UNC, GDAC	hayes@med.unc.edu		Lineberger Comprehensive Cancer Center, UNC Chapel Hill, 450 West Drive. Chapel Hill, NC 27599		
Mr.	Alan P.	Hoyle	UNC, GDAC	alanh@email.unc.edu		Lineberger Comprehensive Cancer Center, University of North Carolina at Chapel Hill, Chapel Hill, NC 27599 USA		
Dr.	Stuart R.	Jeffreys	UNC, GDAC	stuart_jefferys@med.unc.edu		Lineberger Comprehensive Cancer Center, University of North Carolina at Chapel Hill, Chapel Hill, NC 27599 USA		
Mr.	Lisle E.	Mose	UNC, GDAC	lmose@email.unc.edu		Lineberger Comprehensive Cancer Center, University of North Carolina at Chapel Hill, Chapel Hill, NC 27599 USA		
Ms.	Janae V.	Simons	UNC, GDAC	janae_simons@med.unc.edu		Lineberger Comprehensive Cancer Center, University of North Carolina at Chapel Hill, Chapel Hill, NC 27599 USA		
Mr.	Mathew G.	Soloway	UNC, GDAC	msoloway@email.unc.edu		Lineberger Comprehensive Cancer Center, University of North Carolina at Chapel Hill, Chapel Hill, NC 27599 USA		
Dr.	Stephen B.	Baylin	USC/JHU, GCC	sbaylin@jhmi.edu		Cancer Biology Division, The Sidney Kimmel Comprehensive Cancer Center at Johns Hopkins University, Baltimore, Maryland 21287, USA		
Dr.	Benjamin P.	Berman	USC/JHU, GCC	benbfly@gmail.com		USC Epigenome Center, University of Southern California Keck School of Medicine, 1450 Biggy Street, Los Angeles, CA 90033, USA		
Mr.	Moiz S.	Bootwalla	USC/JHU, GCC	msbootwalla@gmail.com		USC Epigenome Center, University of Southern California Keck School of Medicine, 1450 Biggy Street, Los Angeles, CA 90033, USA		
Dr.	Ludmila	Danilova	USC/JHU, GCC	ludmila.danilova@gmail.com		The Sidney Kimmel Comprehensive Cancer Center at Johns Hopkins University, Baltimore, Maryland 21287, USA		
Dr.	James G.	Herman	USC/JHU, GCC	hermaji@jhmi.edu		Cancer Biology Program, The Sidney Kimmel Comprehensive Cancer Center at Johns Hopkins University, Baltimore, Maryland 21287, USA		
Dr.	Toshinori	Hinoue	USC/JHU, GCC	thinoue@usc.edu		USC Epigenome Center, University of Southern California Keck School of Medicine, 1450 Biggy Street, Los Angeles, CA 90033, USA		
Dr.	Peter W.	Laird	USC/JHU, GCC	plaird@usc.edu		USC Epigenome Center, University of Southern California Keck School of Medicine, 1450 Biggy Street, Los Angeles, CA 90033, USA		
Dr.	Suhn K.	Rhie	USC/JHU, GCC	rhie@usc.edu		USC Epigenome Center, University of Southern California Keck School of Medicine, 1450 Biggy Street, Los Angeles, CA 90033, USA		

Dr.	Hui	Shen	USC/JHU, GCC	huis@usc.edu		USC Epigenome Center, University of Southern California Keck School of Medicine, 1450 Biggy Street, Los Angeles, CA 90033, USA		
Dr.	Timothy	Triche Jr.	USC/JHU, GCC	ttriche@usc.edu		USC Epigenome Center, University of Southern California Keck School of Medicine, 1450 Biggy Street, Los Angeles, CA 90033, USA		
Dr.	Daniel J.	Weisenberger	USC/JHU, GCC	weisenbe@usc.edu		USC Epigenome Center, University of Southern California Keck School of Medicine, 1450 Biggy Street, Los Angeles, CA 90033, USA		
Dr.	Stanley R.	Hamilton	MDACC, GDAC	shamilto@mdanderson.org		Univ. of Texas MD Anderson Cancer Center, 1515 Holcombe Blvd. Houston,Texas		
Mr.	Scott	Abbott	WashU, GSC	sabbott@genome.wustl.edu		The Genome Institute, Washington University, St Louis, MO 63108, USA		
Ms.	Rachel	Abbott	WashU, GSC	rabbott@genome.wustl.edu		The Genome Institute, Washington University, St Louis, MO 63108, USA		
Dr.	Nathan D.	Dees	WashU, GSC	nathanddees@gmail.com		The Genome Institute, Washington University, St Louis, MO 63108, USA		
Ms.	Kim	Delehaunty	WashU, GSC	kdelehau@genome.wustl.edu		The Genome Institute, Washington University, St Louis, MO 63108, USA		
Dr.	Li	Ding	WashU, GSC	lding@genome.wustl.edu		The Genome Institute, Washington University, St Louis, MO 63108, USA	Department of Medicine, Washington University, St Louis, MO 63110, USA	Siteman Cancer Center, Washington University, St Louis, MO 63110, USA
Mr.	David J.	Dooling	WashU, GSC	ddgenome@gmail.com		The Genome Institute, Washington University, St Louis, MO 63108, USA		
Mr.	Jim M.	Eldred	WashU, GSC	jeldred@genome.wustl.edu		The Genome Institute, Washington University, St Louis, MO 63108, USA		
Ms.	Catrina C.	Fronick	WashU, GSC	cfronick@genome.wustl.edu		The Genome Institute, Washington University, St Louis, MO 63108, USA		
Mr.	Robert	Fulton	WashU, GSC	bfulton@genome.wustl.edu		The Genome Institute, Washington University, St Louis, MO 63108, USA		
Ms.	Lucinda L.	Fulton	WashU, GSC	lfulton@genome.wustl.edu		The Genome Institute, Washington University, St Louis, MO 63108, USA		
Ms.	Joelle	Kalicki-Veizer	WashU, GSC	jveizer@genome.wustl.edu		The Genome Institute, Washington University, St Louis, MO 63108, USA		
Ms.	Krishna-Latha	Kanchi	WashU, GSC	kkanchi@genome.wustl.edu		The Genome Institute, Washington University, St Louis, MO 63108, USA		
Dr.	Cyriac	Kandoth	WashU, GSC	ckandoth@gmail.com	cyriac	The Genome Institute, Washington University, St Louis, MO 63108, USA		
Mr.	Daniel C.	Koboldt	WashU, GSC	dkoboldt@genome.wustl.edu		The Genome Institute, Washington University, St Louis, MO 63108, USA		
Dr.	David E.	Larson	WashU, GSC	dlarson@genome.wustl.edu		The Genome Institute, Washington University, St Louis, MO 63108, USA		
Dr.	Timothy J.	Ley	WashU, GSC	timley@wustl.edu		The Genome Institute, Washington University, St Louis, MO 63108, USA	Division of Oncology, Washington University in St. Louis, MO 63110, USA	
Ms.	Ling	Lin	WashU, GSC	llin@genome.wustl.edu		The Genome Institute, Washington University, St Louis, MO 63108, USA		
Dr.	Charles	Lu	WashU, GSC	clu@genome.wustl.edu		The Genome Institute, Washington University, St Louis, MO 63108, USA		
Dr.	Vincent J.	Magrini	WashU, GSC	vmagrini@genome.wustl.edu		The Genome Institute, Washington University, St Louis, MO 63108, USA		
Dr.	Elaine R.	Mardis	WashU, GSC	emardis@wustl.edu		The Genome Institute, Washington University, St Louis, MO 63108, USA	Department of Genetics, Washington University in St. Louis, MO 63108, USA	Siteman Cancer Center, Washington University, St Louis, MO 63110, USA
Mr.	Michael D.	McLellan	WashU, GSC	mmclella@genome.wustl.edu		The Genome Institute, Washington University, St Louis, MO 63108, USA		
Mr.	Joshua F.	McMichael	WashU, GSC	jmcmicha@genome.wustl.edu		The Genome Institute, Washington University, St Louis, MO 63108, USA		
Dr.	Christopher A.	Miller	WashU, GSC	cmiller@genome.wustl.edu		The Genome Institute, Washington University, St Louis, MO 63108, USA		
Ms.	Michelle	O'Laughlin	WashU, GSC	mharriso@genome.wustl.edu		The Genome Institute, Washington University, St Louis, MO 63108, USA		
Mr.	Craig	Pohl	WashU, GSC	cpohl@genome.wustl.edu		The Genome Institute, Washington University, St Louis, MO 63108, USA		
Ms.	Heather	Schmidt	WashU, GSC	hschmidt@genome.wustl.edu		The Genome Institute, Washington University, St Louis, MO 63108, USA		
Mr.	Scott M.	Smith	WashU, GSC	ssmith@genome.wustl.edu		The Genome Institute, Washington University, St Louis, MO 63108, USA		
Mr.	Jason	Walker	WashU, GSC	jwalker@genome.wustl.edu		The Genome Institute, Washington University, St Louis, MO 63108, USA		
Dr.	John W.	Wallis	WashU, GSC	john.w.wallis+retiree@gmail.com		The Genome Institute, Washington University, St Louis, MO 63108, USA		
Dr.	Michael C.	Wendl	WashU, GSC	mwendl@genome.wustl.edu		The Genome Institute, Washington University, St Louis, MO 63108, USA	Department of Genetics, Washington University, St Louis, MO 63110, USA	Department of Mathematics, Washington University, St Louis, MO 63130, USA
Dr.	Richard K.	Wilson	WashU, GSC	rwilson@wustl.edu		The Genome Institute, Washington University, St Louis, MO 63108, USA	Department of Genetics, Washington University in St. Louis, MO 63108, USA	Siteman Cancer Center, Washington University, St Louis, MO 63110, USA
Mr.	Todd	Wylie	WashU, GSC	twylie@genome.wustl.edu		The Genome Institute, Washington University, St Louis, MO 63108, USA		
Dr.	Qunyuan	Zhang	WashU, GSC	qunyuan@wustl.edu		The Genome Institute, Washington University, St Louis, MO 63108, USA	Department of Genetics, Washington University, St Louis, MO 63110, USA	

Salutation	First Name	Last Name	Email Address	Nature ID (optional)	Contribution	Institution #1 (address, zip code)	Institution #2 (address, zip code)	Institution #3 (address, zip code)	Comments
Dr.	Larsson	Omberg	larsson.omberg@sagebase.org		Pan-Can Data freeze	Sage Bionetworks 1100 Fairview Ave North, M1-C108, 98109-1024			
Dr.	Adam	Margolin	margolin@sagebase.org		Pan-Can Data freeze	Sage Bionetworks 1100 Fairview Ave North, M1-C108, 98109-1024			
Dr.	Benjamin J.	Raphael	braphael@brown.edu	braphael	AWG	Department of Compuer Science & Center for Computational Molecular Biology, Brown University, 115 Waterman St, Providence RI 02912			
Dr.	Fabio	Vandin	vandinfa@cs.brown.edu		AWG	Department of Compuer Science & Center for Computational Molecular Biology, Brown University, 115 Waterman St, Providence RI 02912			
Mr.	Hsin-Ta	Wu	bournewu@cs.brown.edu		AWG	Department of Compuer Science & Center for Computational Molecular Biology, Brown University, 115 Waterman St, Providence RI 02912			
Mr.	Mark D. M.	Leiserson	mdml@cs.brown.edu		AWG	Department of Compuer Science & Center for Computational Molecular Biology, Brown University, 115 Waterman St, Providence RI 02912			
Dr.	Stephen C.	Benz	steve@five3genomics.com		AWG (pan-cancer)	Five3 Genomics LLC, 101 Cooper St, Santa Cruz, CA 95060			
Dr.	Charles J.	Vaske	charlie@five3genomics.com		AWG (pan-cancer)	Five3 Genomics LLC, 101 Cooper St, Santa Cruz, CA 95060			
Dr.	Houtan	Noushmehr	houtan@usp.br		AWG (GBM & LGG)	Department of Genetics, Ribeirão Preto Medical School, University of São Paulo, São Paulo, Brazil	Center for Integrative Systems Biology - CISBi, NAP/USP, São Paulo, Brazil		
Dr.	Theo	Knijnenburg	tknijnen@systemsbiology.org		AWG	Institute for Systems Biology, 401 Terry Avenue North, Seattle, WA 98109			
Dr.	Denise	Wolf	Denise.Wolf@ucsf.edu		AWG (pan-cancer)	Department of Laboratory Medicine, University of California San Francisco, 2340 Sutter St, San Francisco, CA, 94115			
Dr.	Laura	Van 't Veer	VantveerL@cc.ucsf.edu		AWG (pan-cancer)	Department of Laboratory Medicine, University of California San Francisco, 2340 Sutter St, San Francisco, CA, 94115			
Dr.	Eric	Collisson	eric.collisson@ucsf.edu	ecollisson	AWG (pan-cancer)	Department of Medicine, University of California San Francisco, 450 35d St, San Francisco, CA, 94148			
Dr.	Dimitris	Anastassiou	da8@columbia.edu		AWG (Pan-Cancer)	Department of Electrical Engineering, Columbia University, New York, NY 10027			
Mr.	Tai-Hsien	Ou Yang	to2232@columbia.edu		AWG (Pan-Cancer)	Department of Electrical Engineering, Columbia University, New York, NY 10027			
Dr	Nuria	Lopez-Bigas	nuria.lopez@upf.edu		AWG (Pan-Cancer)	Research Unit on Biomedical Informatics, Department of Experimental and Health Sciences, Universitat Pompeu Fabra, Dr. Aiguader 88, Barcelona, Spain	Catalan Institution for Research and Advanced Studies (ICREA), Passeig Lluís Companys, 23, Barcelona, Spain		
Dr	Abel	Gonzalez-Perez	abel.gonzalez@upf.edu		AWG (Pan-Cancer)	Research Unit on Biomedical Informatics, Department of Experimental and Health Sciences, Universitat Pompeu Fabra, Dr. Aiguader 88, Barcelona, Spain			
Dr	David	Tamborero	david.tamborero@upf.edu		AWG (Pan-Cancer)	Research Unit on Biomedical Informatics, Department of Experimental and Health Sciences, Universitat Pompeu Fabra, Dr. Aiguader 88, Barcelona, Spain			
Dr.	Zheng	Xia	zxia@bcm.edu		AWG (Pan-Cancer)	Dan L Duncan Cancer Center, Baylor College of Medicine, One Baylor Plaza, Houston, TX, 77030			
Dr.	Wei	Li	WL1@bcm.edu		AWG (Pan-Cancer)	Dan L Duncan Cancer Center, Baylor College of Medicine, One Baylor Plaza, Houston, TX,			
Dr.	Dong-Yeon	Cho	chody@ncbi.nlm.nih.gov		Subtyping (Pan-Cancer)	National Center for Biotechnology Information, National Library of Medicine, National Institutes of Health, 8600 Rockville Pike, Bethesda, MD 20894			Please include statement: Supported by Intramural Research program of NLM, NIH
Dr.	Teresa	Przytycka	przytyck@ncbi.nlm.nih.gov		Subtyping (Pan-Cancer)	National Center for Biotechnology Information, National Library of Medicine, National Institutes of Health, 8600 Rockville Pike, Bethesda, MD 20894			Please include statement: Supported by Intramural Research program of NLM, NIH
Mr.	Mark	Hamilton	mphamilt@bcm.edu		miRNA/non-coding RNA analysis	Department of Molecular and Cellular Biology, Baylor College of Medicine, One Baylor Plaza, Houston, TX, 77030			
Dr.	Sean	McGuire	semcguir@bcm.edu		miRNA/non-coding RNA analysis	Department of Molecular and Cellular Biology, Baylor College of Medicine, One Baylor Plaza, Houston, TX, 77030			
Dr	Sven	Nelander	sven.nelander@igp.uu.se		Integrative modeling and validation of candidate genes by siRNA (pancan)	Uppsala University, Institution for Immunology, Genetics and Pathology and Science for Life Laboratory; formerly computational biology center at MSKCC			
Mr	Patrik	Johansson	patrik.johansson@igp.uu.se		Integrative modeling and validation of candidate genes by siRNA (pancan)	Uppsala University, Institution for Immunology, Genetics and Pathology and Science for Life Laboratory			
Dr	Rebecka	Jörnsten	jornsten@chalmers.se		Integrative modeling and validation of candidate genes by siRNA (pancan)	Chalmers Technical University, Mathematical sciences			

Ms	Teresia	Kling	teresia.kling@gu.se	Integrative modeling and validation of candidate genes by siRNA (pancan)	Uppsala University, Institution for Immunology, Genetics and Pathology and Science for Life Laboratory
Mr	Jose	Sanchez	sanchesj@chalmers.se	optimization methods of integrative modeling of pancan data	Chalmers Technical University, Mathematical sciences

GCC	Genome Characterization Center
GDAC	Genome Data Analysis Center
GSC	Genome Sequencing Center
DCC	Data Coordinating Center
TSS	Tissue Source Site
BCR	Biospecimen Core Resource Center
AWG	Analysis Working Group
Link to TCGA components page:	http://cancergenome.nih.gov/abouttcga/overview
