# Supplementary material for: The mutational landscape of phosphorylation signaling in cancer
Source: Sci Rep. 2013 Oct 2;3:2651. doi: 10.1038/srep02651 (PMC3788619; doi:10.1038/srep02651)
Supplement: Supplementary Information [file srep02651-s3.pdf]

SUPPLEMENTARY INFORMATION
The mutational landscape of phosphorylation signaling in cancer

Jüri Reimand*, Omar Wagih, Gary D. Bader*

The Donnelly Centre, University of Toronto, Canada

* Correspondence: Gary.Bader@utoronto.ca or Juri.Reimand@utoronto.ca

SUPPLEMENTARY INFORMATION: Table of contents

Supplementary Table 1. Sample and mutation counts for cancer types. **3**

Supplementary Figure 1. Global distribution of mutations in phosphosites. **4**

Supplementary Figure 2. Distribution of disordered and structured phosphosites. **5**

Supplementary Figure 3. Binding specificity models (PWMs) for 96 kinases. **6**

Supplementary Figure 3 (continued). **7**

Supplementary Figure 4. Top mutated genes from pSNV pathway analysis. **8**

Supplementary Note 1. Descriptions of Supplementary Tables provided separately. **9**

code		cancer type	#samples	#SNV	#pSNV
blca		bladder urothelial carcinoma	98	17,143	1,400
brca		breast invasive carcinoma	761	23,989	1,815
coadread		colon,rectum adenocarcinoma	193	12,500	783
gbm		glioblastoma multiforme	289	13,236	934
hnsc		head and neck squamous cell carcinoma	301	31,577	2,261
kirc		kidney renal clear cell carcinoma	417	15,610	1,154
laml		acute myeloid leukemia	184	1,383	157
luad		lung adenocarcinoma	225	49,333	3,036
lusc		lung squamous cell carcinoma	172	36,724	2,339
ov		ovarian serous cystadenocarcinoma	316	12,111	915
ucec		uterine corpus endometrioid carcinoma	229	28,095	2,046

Supplementary Table 1. Sample and mutation counts for cancer types.

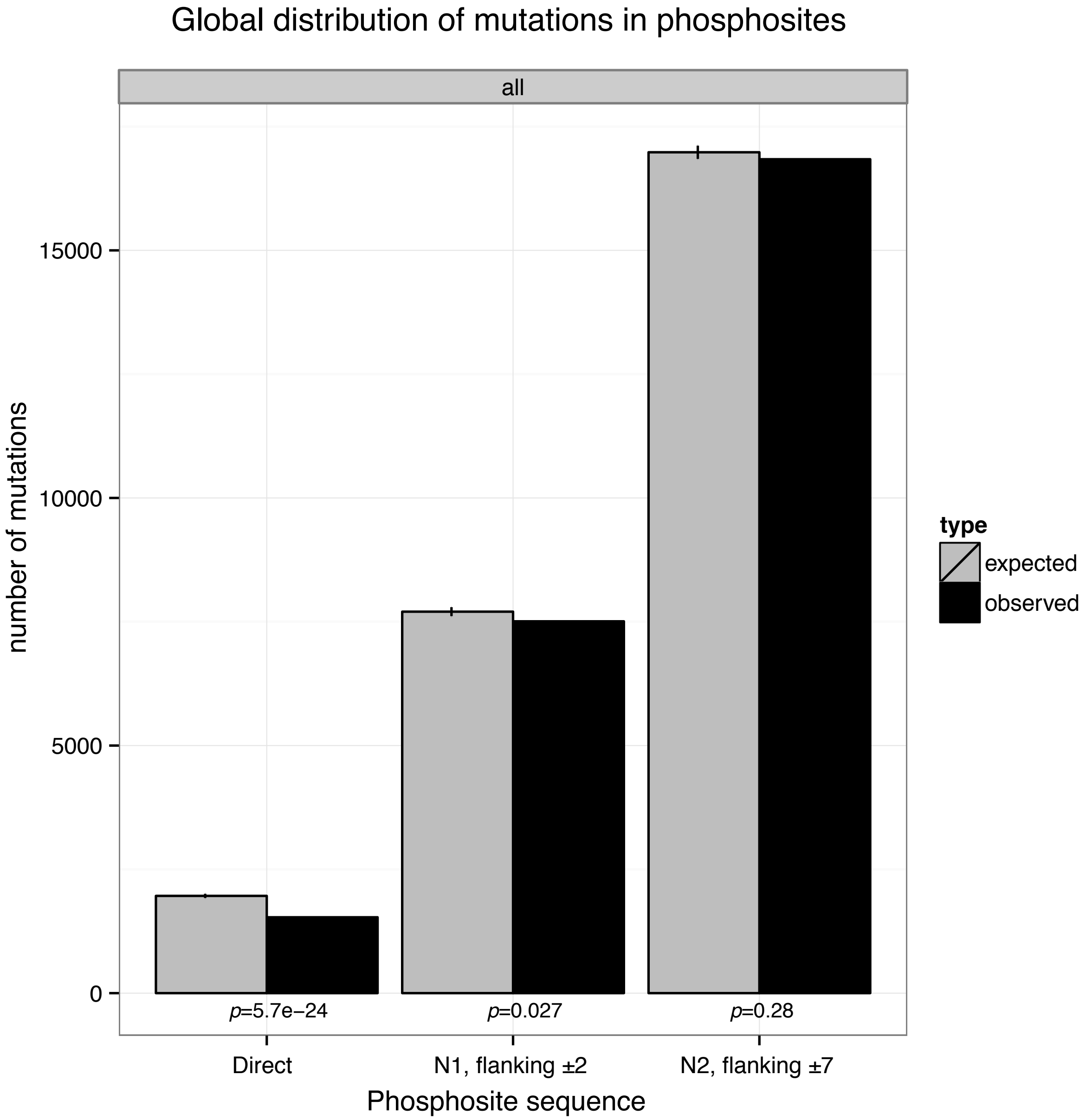

Supplementary Figure 1. Global distribution of mutations in phosphosites. Observed and expected numbers of SNVs in phosphorylated residues (direct mutations) and phosphosite flanking regions. P-values are computed with the two-sided Poisson exact test and shown below barplots. Expected values represent medians sampled from the Poisson distribution, and errorbars denote ±1 median absolute deviation.

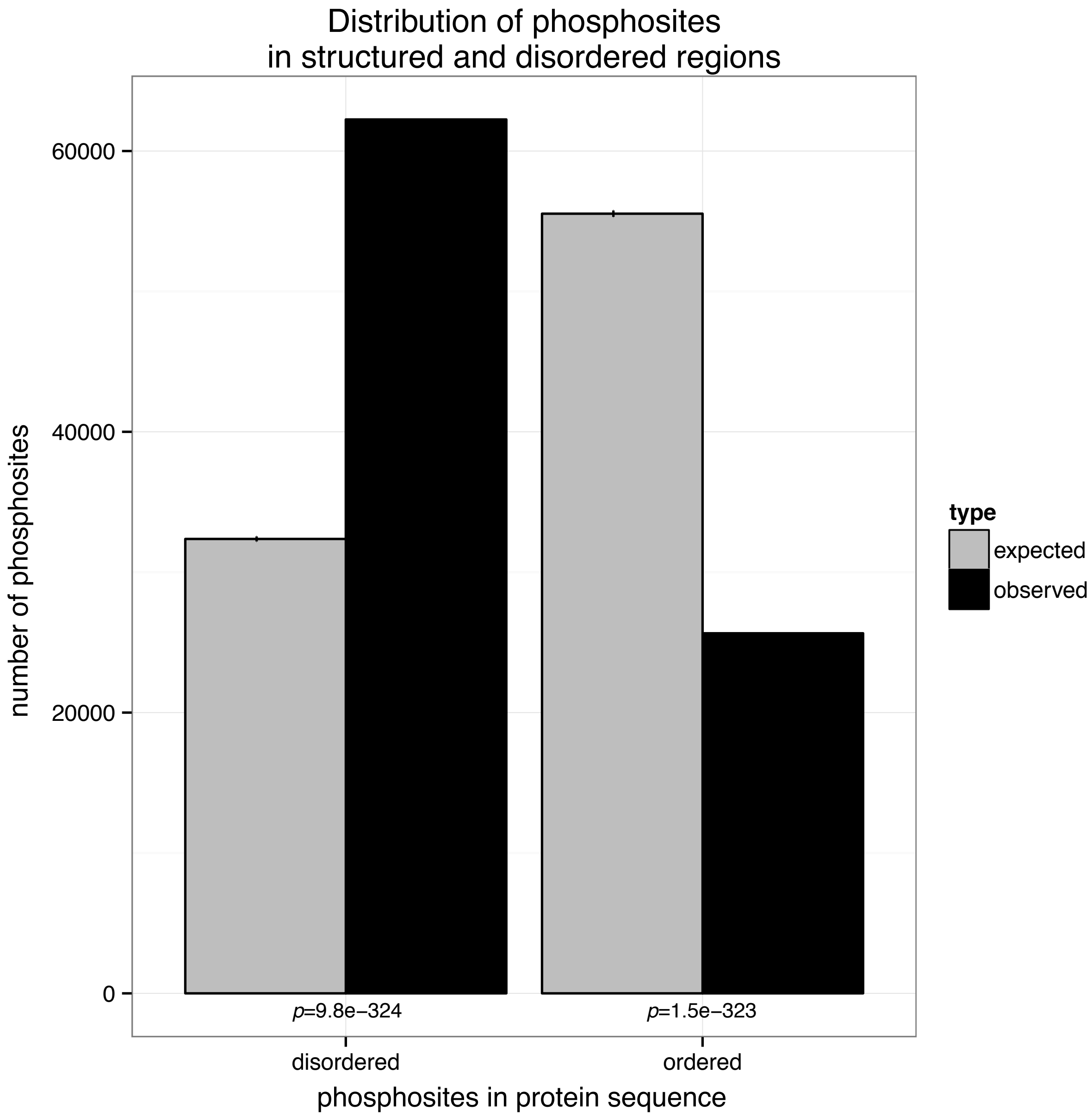

Supplementary Figure 2. Distribution of disordered and structured phosphosites. Disordered regions predicted by DISOPRED2 software (37% of protein sequence) have significantly more phosphosites (black) than expected (gray). P-values are computed with the two-sided Poisson exact test and shown below barplots. Expected values represent medians sampled from the Poisson distribution, and errorbars denote ±1 standard error (median absolute deviation).

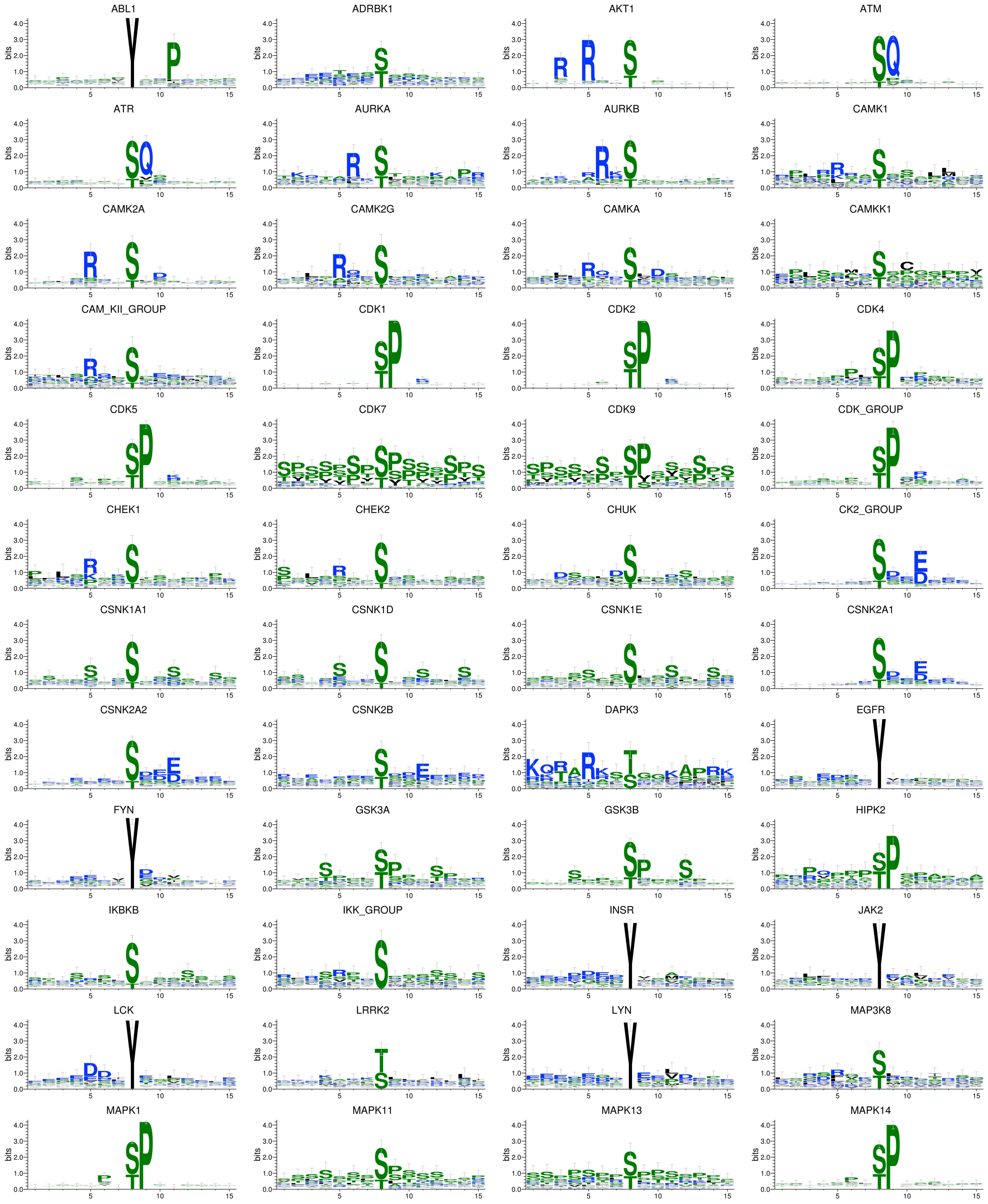

Supplementary Figure 3. Binding specificity models (PWMs) for 96 kinases. X-axis shows binding sequence with central phosphorylated residue at position 8 and flanking sequence of ±7 residues. Y-axis shows information content, and letter height shows relative importance of corresponding amino acid at that position.

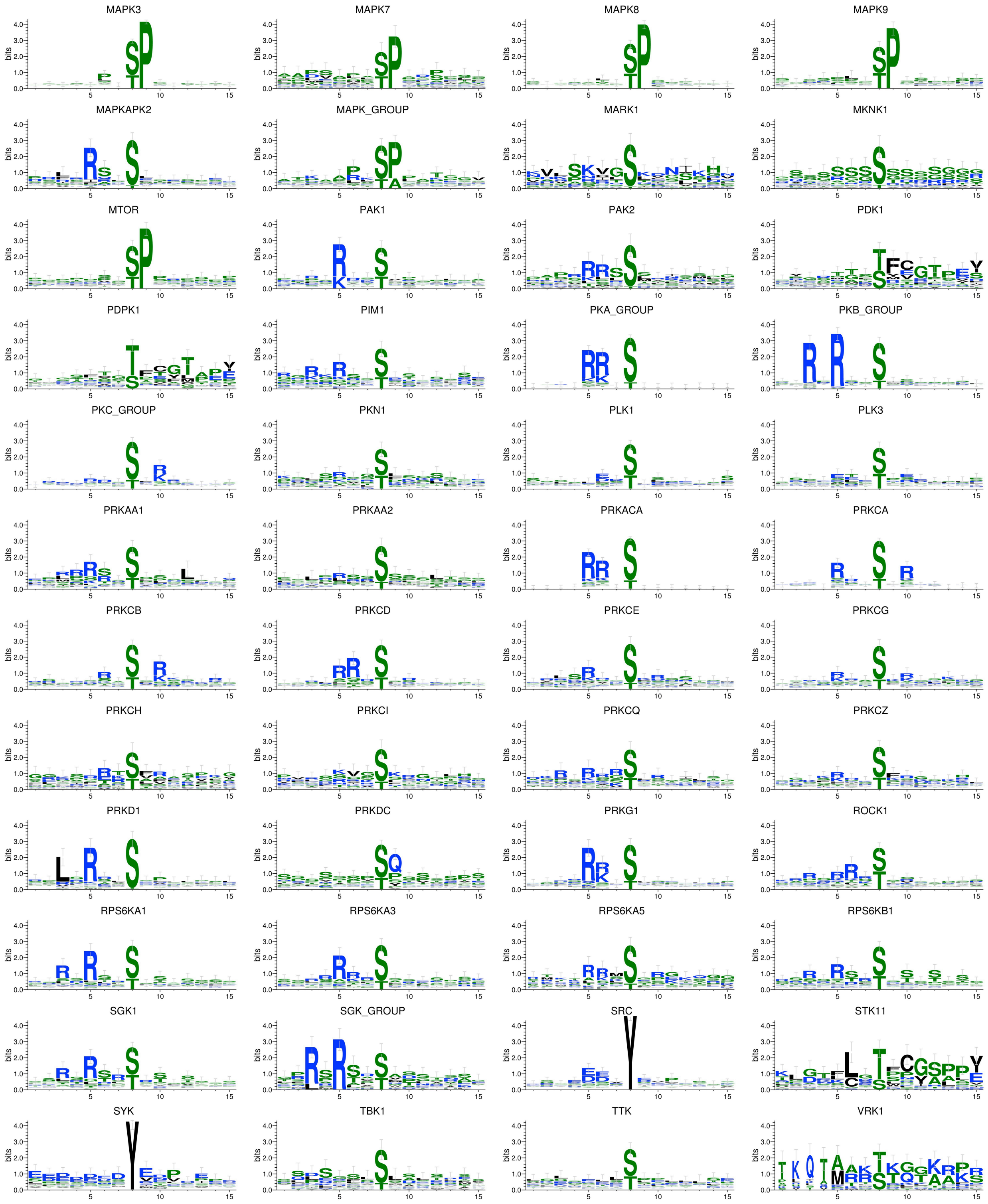

Supplementary Figure 3 (continued).

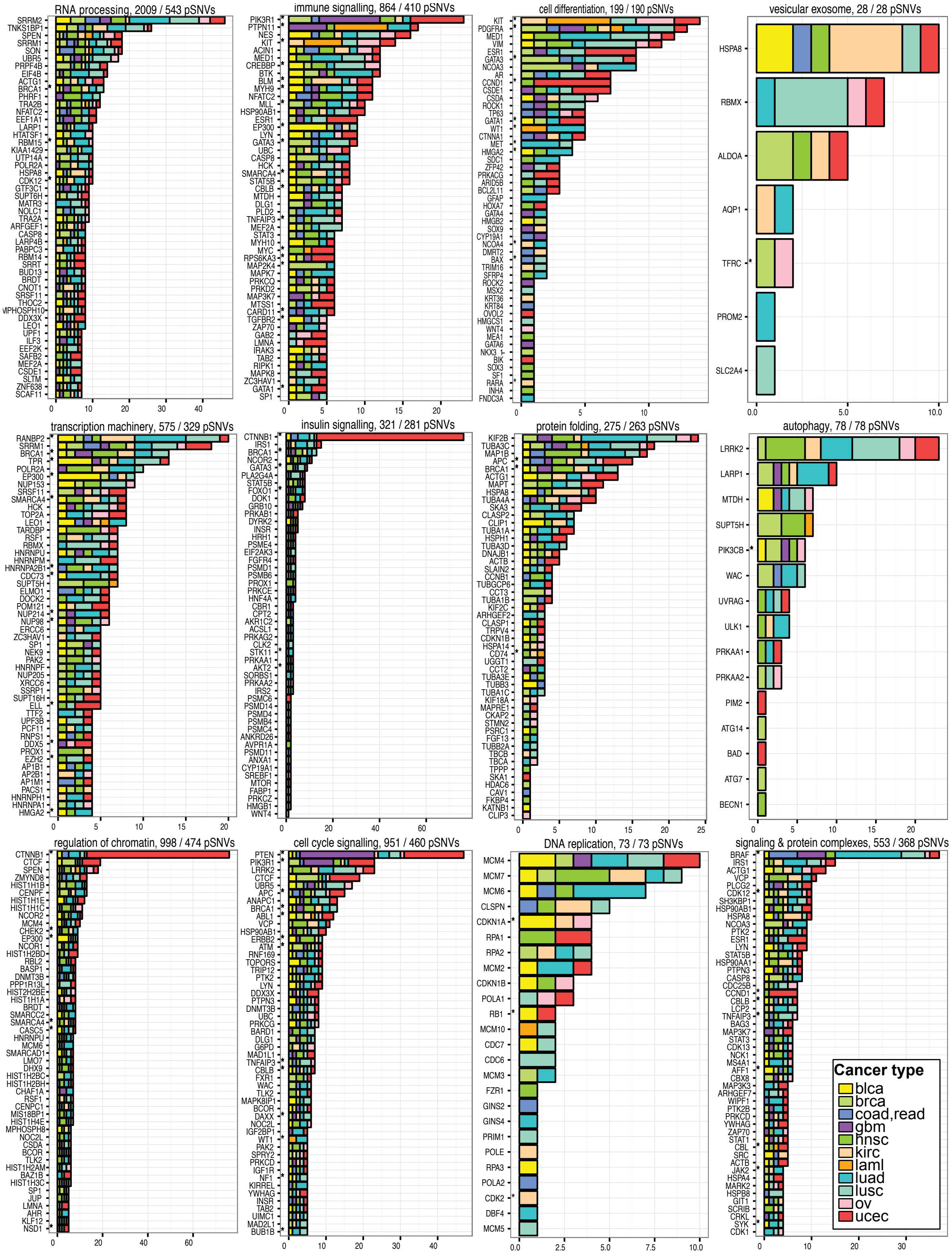

Supplementary Figure 4. Top mutated genes from pSNV pathway analysis.
Top-50 most frequently mutated genes of each functional theme from pSNV-specific pathway enrichment analysis. Asterisks denote known cancer genes. Total and top-50 count of pSNVs per theme is shown in panel title.

Supplementary Note 1.
Descriptions of Supplementary Tables provided separately.

Supplementary Table 2. Phosphosites with somatic mutations in pan-cancer genomes. Shown are phosphosites with at least one flanking mutation within ±7 residues. Mutation counts in columns DI_*pSNV_count* (direct), *N1_pSNV_count* (±2 flanking sequence), and *N2_pSNV_count* (±7 flanking sequence) are partially redundant as pSNVs frequently affect multiple sites. The column *pmid* shows PubMed IDs of associated phosphoproteomics publications, and "PSP" in column *pmid* means that references are available in the online version of PhosphositePlus database (http://www.phosphosite.org). The column *disorder* shows if the phosphosite is predicted to be in disordered (1) or structured (0) protein sequence. The column *peptide15* includes the phosphorylated site shown in lowercase an ±7 of residues of associated flanking sequence. Shorter sequences denote sites in protein termini, where carat (^) denotes N-terminus and asterisk (*) denotes C-terminus.
Supplementary Table 3. Phosphorylation-associated single nucleotide variants (pSNVs). Shown are all somatic mutations in pan-cancer genomes with a phosphosite within ±7 residues. The column *status* shows distance of pSNV to nearest phosphosite (DI - direct mutation of phosphorylated residue; N1 - phosphosite within ±2 residues; N2 - phosphosite within ±7 residues). Cancer type-specific pSNVs are shown in columns **_counts*, and *total_count* shows the total number of pSNVs in pan-cancer genomes.
Supplementary Table 4. ActiveDriver predictions of genes with significant pSNVs. Shown are all significant genes from the integrated analysis of pan-cancer data as well as analyses of individual cancer types (p<0.01). Cancer type is shown in the column *cancer_type*, and rows with *cancer_type* "PAN" include pan-cancer predictions.
Supplementary Table 5. Mutations and phosphosites corresponding to network rewiring events. Shown are all significant network rewiring (p<0.05). The column *position* indicates position of mutation, and its location relative to phosphosite is shown in column *mut_dist_from_psite*. Flanking sequences in columns *flank_wt* and flank_mut include phosphorylated residue in lowercase. Stop codons are shown by asterisks, and phosphosites truncated by protein termini are concatenated with dashes (-). The columns *wt_score*, *mut_score* include PWM matching scores to wildtype and mutated sequences, and the columns *wt_pval*, *mut_pval* include corresponding p-values.
Supplementary Table 6. Functional enrichment analysis of pSNVs. Shown are Gene Ontology terms, pathways and protein complexes specifically enriched in pan-cancer phosphosite mutations (p<0.01). Functional terms also enriched in global SNVs have been filtered.
Supplementary Table 7. Domains with enriched pSNVs. Shown are non-filtered protein domains with enriched pSNVs. The column *domain_id* contains domain identifiers in Pfam and SMART databases (Pfam entries are prefixed). The column *domain_pSNVs* includes genes and positions of domain-specific phosphosite mutations.
Supplementary Table 8. Protein-protein interaction network of proteins with domain-specific pSNVs. Shown are physical interactions between proteins with enriched domain-specific pSNVs. Domains are grouped manually as kinases, phosphatases, histones, transcription factors and RAS proteins.
